# Genome-wide identification and expression analysis of the WRKY gene family in *Rhododendron henanense* subsp. *lingbaoense*

**DOI:** 10.7717/peerj.17435

**Published:** 2024-05-29

**Authors:** Xiangmeng Guo, Xinyu Yan, Yonghui Li

**Affiliations:** School of Life Sciences, Luoyang Normal University, Luoyang, Henan, China

**Keywords:** *Rhododendron henanense* subsp. *Lingbaoense* (Rhl), WRKY, Bioinformatics, Abiotic stress, Expression patterns

## Abstract

**Background:**

This work explored the characteristics of the WRKY transcription factor family in *Rhododendron henanense* subsp. *lingbaoense* (*Rhl*) and the expression patterns of these genes under abiotic stress by conducting bioinformatics and expression analyses.

**Methods:**

*RhlWRKY* genes were identified from a gene library of *Rhl*. Various aspects of these genes were analyzed, including genetic structures, conserved sequences, physicochemical properties, *cis*-acting elements, and chromosomal location. RNA-seq was employed to analyze gene expression in five different tissues of *Rhl*: roots, stems, leaves, flowers, and hypocotyls. Additionally, qRT-PCR was used to detect changes in the expression of five *RhlWRKY* genes under abiotic stress.

**Result:**

A total of 65 *RhlWRKY* genes were identified and categorized into three subfamilies based on their structural characteristics: Groups I, II, and III. Group II was further divided into five subtribes, with shared similar genetic structures and conserved motifs among members of the same subtribe. The physicochemical properties of these proteins varied, but the proteins are generally predicted to be hydrophilic. Most proteins are predicted to be in the cell nucleus, and distributed across 12 chromosomes. A total of 84 *cis*-acting elements were discovered, with many related to responses to biotic stress. Among the identified *RhlWRKY* genes, there were eight tandem duplicates and 97 segmental duplicates. The majority of duplicate gene pairs exhibited Ka/Ks values <1, indicating purification under environmental pressure. GO annotation analysis indicated that WRKY genes regulate biological processes and participate in a variety of molecular functions. Transcriptome data revealed varying expression levels of 66.15% of WRKY family genes in all five tissue types (roots, stems, leaves, flowers, and hypocotyls). Five *RhlWRKY* genes were selected for further characterization and there were changes in expression levels for these genes in response to various stresses.

**Conclusion:**

The analysis identified 65 *RhlWRKY* genes, among which the expression of *WRKY_42* and *WRKY_17* were mainly modulated by the drought and MeJA, and *WRKY_19* was regulated by the low-temperature and high-salinity conditions. This insight into the potential functions of certain genes contributes to understanding the growth regulatory capabilities of *Rhl*.

## Introduction

*Rhododendron henanense* subsp. *lingbaoense* (hereinafter referred to as *Rhl*) is a subspecies of *Rhododendron henanense* native to the western part of Henan Province, concentrated within the Xiaoqinling National Nature Reserve (Yue, Han & Zhang, 2019). *Rhl* is known for its vibrant and clustered blossoms, making it a highly valued ornamental plant. However, its habitat is currently in decline, resulting in a rapid decrease in plant numbers, putting this subspecies in a precarious state. As a unique species, *Rhl* is a valuable plant to study with significant natural heritage value and the added importance of being a repository of genetic resources (Zhou et al., 2019). In recent years, many scholars, both domestically and internationally, have shown considerable interest in studying the medicinal ingredients of different *Rhododendron* species, such as the inhibition of liver cancer cell activity by lupeol and uvaol from *Rhododendron micranthum* (Chang, 2011) and the treatment of vascular inflammation by hyperoside from *Rhododendron brachycarpum* G. Don (Ku et al., 2015). Overall, components of *Rhododendron* species hold potential for various applications, including ecological conservation, aesthetics, medicinal uses, and scientific research (Li et al., 2018; Liang et al., 2016) Plants continuously face various stressors, during their growth potentially limiting their growth and development (Chen et al., 2012). At the molecular level, the induction of stress resistance genes helps plants adapt to unfavorable environmental conditions (Matsui et al., 2008). Among these genes, WRKY transcription factors are part of a large family of plant regulatory proteins. The most distinctive feature of WRKY transcription factors is a DNA-binding domain composed of approximately 60 amino acids, with a highly conserved heptapeptide sequence WRKYGQK at the N-terminus (Jiang et al., 2015), which gives this family its name. WRKY transcription factors are classified into three major categories, WRKY I, II, and III, based on differences in their conserved domains. WRKY I possesses two WRKY conserved domains with a C2H2 zinc finger, WRKY II contains only one WRKY conserved domain with a C2H2 zinc finger, and WRKY III has one WRKY conserved domain with a C2HC zinc finger (Vives-Peris et al., 2018). The regulation of gene expression by WRKY transcription factors primarily occurs through binding to specific *cis*-regulatory elements called W-box (TTGACC) elements. This binding can activate or suppress the transcription of downstream target genes and is also subject to regulation by upstream master regulatory proteins (Huang et al., 2019). WRKY transcription factors play crucial roles in plant growth, development, and responses to abiotic stresses. For instance, WRKY transcripts were expressed in elongating fiber ovules in cotton three days after flowering, suggesting involvement of these transcription factors in fiber development (Wang et al., 2010). *WRKY* genes also play vital roles in the development of plant anthers and embryos (Zhang et al., 2018). *GhWRKY33* in upland cotton responds to drought stress and significantly enhances drought resistance when overexpressed in transgenic *Arabidopsis* (Wei et al., 2020). Overexpression of the rice gene *OsWRKY42* reduces cell wall damage caused by high-salinity stress, ultimately enhancing salt tolerance (Pillai et al., 2018). Under low-temperature stress, overexpression of *PmWRKY40* gene in plum enhances the cold resistance of plum flowers (Peng et al., 2019).

The first *WRKY* gene family member, *SPF I*, was discovered in sweet potato in 1994 and found to be induced by sucrose and PAG (Bu et al., 2020). Subsequently, *WRKY* genes have been identified in many plant genomes, including *Arabidopsis* (Eulgem et al., 2000), sweet orange (Silva, Ito & Souza, 2017), sesame (Li et al., 2017), cassava (Wei et al., 2016) and maize (Hu et al., 2021). Recent studies of *Rhl* have mainly focused on genetic evolution, the development of ornamental aspects, and predictions of *MYB* gene family functions (Yue, Han & Zhang, 2019; Zhou et al., 2019, 2022; Han et al., 2008). However, there has been no study of the *WRKY* gene family in this plant. This work used bioinformatics analysis to identify the *WRKY* genes in *Rhl* and investigated their expression patterns in different tissues and under abiotic stress conditions. The results lay the foundation to explore the biological functions of *WRKY* genes in this important sub-species.

## Materials and Methods

### Prediction of physicochemical properties

The physicochemical properties of the *RhlWRKY* protein were predicted using the compute PI/MW tool of ExPASy database (http://web.expasy.org/compute_pi/), and subcellular localization was predicted using the WoLFPSORT website (https://www.genscript.com/wolf-psort.html).

### Gene identification and phylogenetic tree construction

Firstly, InterProScan (v5.50-84.0) was used to annotate the RhlWRKY protein domain based on InterPro protein database, and the annotation results of pfam gene family were extracted. Other transcription factor families not annotated by InterPro are annotated with PlantTFDB (https://planttfdb.gao-lab.org/). A total of 65 putative *WRKY* genes were identified, and identify the candidate *RhlWRKY* from the genome of *Rhl* (SAR: PRJNA656593) (Zhou et al., 2022). Next, MEGA7.0 was used to conduct cluster analysis of the 90 Arabidopsis protein sequences and the 65 putative *RhlWRKY* proteins to compare their evolutionary with 1,000 replicates bootstrap analysis for statistical reliability. We further performed a NJ phylogenetic tree of the whole *RhlWRKY* protein sequences relationships.

### Gene structure and conserved motif analysis

A phylogenetic tree was constructed using MEGA7.0, and then the RhlWRKY Protein sequence was used to find conserved motifs by using the Multiple Em for MEME online website (https://meme-suite.org/meme/) with zero and one per sequence, maximum number of motifs sets at 12 and optimum width of motif from 6 to 200. The motif with an e-value less than 1e−10 was retained for further analysis. The genetic structure was determined for the *RhlWRKY* genes based on the genome Gff3 annotation file. The Gene Structure View (Advanced) software of the TBtools (v2.038) was used to create a phylogenetic tree, motif, and genetic structure diagrams.

### *Cis*-acting elements

The 2,000 bp upstream sequences of the start codons of *RhlWRKY* genes were collected to obtain the promoter sequences using the GXF Sequences Program of the TBtools. Extract of the and analyzed using the PlantCARE database (Lescot et al., 2002) to predict potential *cis*-acting elements.

### Chromosomal location and collinearity analysis

A chromosomal location map was generated using TBtools and collinearity analysis was performed using McScanX software. The BLAS-BLAST GUI Wrapper algorithm was used for Rhl protein internal self-comparison, the Text Marge for MCscanX for regularizing the gff format file, and the Quick Run MCScanX Wrapper algorithm for self-comparison. Repeat events and collinearity relationships of the *RhlWRKY* genes were analyzed. The Ka/Ks values for duplicate *RhlWRKY* gene pairs were calculated using KaKs_Calculator2.0.

### Gene ontology functional annotation

Gene Ontology (GO) annotations for each *RhlWRKY* gene were obtained using Blast2GO software by aligning between the WRKY protein sequences and the NCBI non redundant protein (Conesa et al., 2005). Subsequently, WEGO software (https://wego.genomics.cn/) was used for GO functional classification and to determine gene function distribution (Zhang & Wang, 2005).

### Stress treatments and transcriptomic analysis

Seeds of *Rhl* were collected from Xiaoqinling, Lingbao City, Henan Province. The seeds were decontaminated, sterilized and cultured on MS medium at 25 °C (16 h light/8 h dark). Two-month-old seedlings were subjected to treatments with low-temperature (4 °C), 20% PEG, NaCl (200 mM), and MeJA (200 µM) solutions. Samples were collected at 0, 1, 3, 6, and 12 h with light after treatment and stored at −80 °C. Transcriptome analysis was conducted in May 2023, using material from the hypocotyls of 14-day-old seedlings, as well as roots, stems, leaves, and fully bloomed flowers from three-month-old tissue-cultured plants. Samples were stored at −80 °C for transcriptome sequencing, which was performed in triplicate (three biological and three technical replicates; sequencing services provided by Wuhan Frasergen Bioinformatics Co., Ltd., Wuhan, China). Using SOAPnuke software (v2.1.0) to filter Raw reads, high-quality Clean reads are obtained by removing paired reads containing connectors, reads with a N ratio greater than 0.5%, and reads with a mass value Q ≤ 20 and base number accounting for more than 50% of the entire read. Clean reads were compared with the reference genome using HISAT (v2.2.1) software. The prediction of new transcriptomes was performed using StringTie v1.3.4d software, using the Fragments Per Kilobase of exon model per million mapped fragments values (FPKM values) as an indicator of gene expression level. The distribution of reads on the reference genome generally shows only the distribution of the first 25 longest chromosomes, or Scaold (Fig. S1). DESeq v1.22.2 was used to calculate FPKM ratio (FC, Fold change) and False discovery rate (FDR, False discovery rate) between the difference comparison samples. |log2FC| ≥ 1 and FDR < 0.05 were used as DEGs screening criteria. The statistical power of this experimental design, calculated in RNA SeqPower was shown in Table S1. A transcriptional pattern heatmap of the *RhlWRKY* gene family was generated using the HeatMap feature in TBtools software (with log2(FPKM) Col Scale settings). The RNA-seq data could be searched from the Biosample with accession numbers SAMN37367964–37367966 (root), SAMN37367967–37367969 (stem), SAMN37367970–37367972 (leaf), SAMN37367973–37367975 (hypocotyl), SAMN37367976–37367978 (flower).

### qRT-PCR treatments

RNA was extracted using a magnetic bead total RNA kit from Shanghai Lingjun Biotechnology Co., Ltd., Shanghai, China. The concentration of total RNA and OD260/OD280 values were determined using an ultramicro spectrophotometer (NanoDrop One, Thermo Scientific, Waltham, MA, USA). Electrophoresis was used to identify the integrity of RNA. This RNA was used to synthesize cDNA by reverse transcription. Primers for five genes (see Table S2) were designed using GenScript (https://www.genscript.com) software and synthesized by Sangon Biotech (Shanghai) Co., Ltd., Shanghai, China. The EF1α gene was used as a reference gene. The cDNA from *Rhl* plants treated with NaCl, PEG, MeJA, and low-temperature treatments was used in qPCR reactions. Relative expression levels were calculated using the 2^−ΔΔCt^ method. Each response had a negative control group and contained three biological and three technical replicates. Actin gene (*EF1α*) was used as internal reference gene. Results of NTC was not detected. The obtained results were inputted into GraphPadPrism (9.3) software (grouped –mean set-summary data-separated bar graph) to plot expression model diagram, and one-way ANOVA test was used to assess differences by SPSS (13.0) (Analyze–Compare Means-One-way ANOVA), and the *P*-value of pairwise comparison was obtained by LSD test.

## Results and analysis

### Characteristics analysis

The results, as shown in Table 1, indicate that the 65 *RhlWRKY* genes (*RhlWRKY_1*- *RhlWRKY_65*) exhibit significant variations in the number of encoded amino acids, ranging from 112 amino acids (*RhlWRKY_18*) to 1,037 amino acids (*RhlWRKY_41*). The molecular weight of RhlWRKY proteins ranges from 13.0801 kDa (*RhlWRKY_41*) to 114.6188 kDa (*RhlWRKY_18*), and their isoelectric points (pI) range from 4.39 (*RhlWRKY_34*) to 9.87 (*RhlWRKY_41*), with an average pI of 6.96. Among these, 26 are alkaline (pI > 7) and 39 are acidic (pI < 7). The hydrophilic coefficients are <0 for all RhlWRKY proteins, indicating all RhlWRKY proteins are hydrophilic proteins. Subcellular localization analysis reveals that the majority of these proteins are predicted to be localized in the cell nucleus, with only a few (*RhlWRKY_13, _18, _19, _56, _64*, and *_65*) predicted to be in the chloroplast and cytoplasm. The results revealed that these newly-identified RhlWRKY proteins in *Rhododendron henanense* exhibited a various subcellular distribution, which may be associated with functional diversification in abiotic stress responses. The similar observations for WRKY proteins had been reported in maize (Hu et al., 2021).

**Table 1:** Physicochemical properties of RhlWRKY proteins.

Gene ID	Subcellular localization	Number of amino acids	Relative molecular mass	Isoelectric point (PI)	Hydrophilic coefficient	Instability index	Liposoluble index
*RhlWRKY_1*	Nucleus	382	41,382.10	6.35	−0.58	48.36	63.90
*RhlWRKY_2*	Nucleus	323	35,201.69	6.32	−0.87	68.79	40.77
*RhlWRKY_3*	Nucleus	286	32,564.43	4.65	−1.25	77.41	44.62
*RhlWRKY_4*	Nucleus	281	30,926.61	7.07	−0.66	45.79	68.36
*RhlWRKY_5*	Nucleus	342	38,342.63	9.82	−0.78	49.78	67.54
*RhlWRKY_6*	Nucleus	331	36,235.21	9.73	−0.55	40.86	66.31
*RhlWRKY_7*	Nucleus	572	62,278.99	8.28	−0.72	61.38	57.50
*RhlWRKY_8*	Nucleus	331	36,631.75	5.99	−0.61	54.26	65.68
*RhlWRKY_9*	Nucleus	154	16,842.06	9.30	−0.61	40.57	70.26
*RhlWRKY_10*	Nucleus	318	34,741.16	9.20	−0.57	45.59	66.23
*RhlWRKY_11*	Nucleus	499	54,308.54	5.19	−0.89	56.01	52.99
*RhlWRKY_12*	Nucleus	519	56,199.86	5.22	−0.81	45.12	56.88
*RhlWRKY_13*	Chloroplast	310	34,423.75	5.15	−0.31	59.25	75.45
*RhlWRKY_14*	Nucleus	562	61,577.10	8.58	−0.96	67.04	47.38
*RhlWRKY_15*	Nucleus	312	33,955.31	6.51	−0.89	58.99	43.53
*RhlWRKY_16*	Nucleus	273	30,816.56	8.74	−0.72	53.25	54.32
*RhlWRKY_17*	Nucleus	558	60,808.24	5.36	−0.42	48.65	72.22
*RhlWRKY_18*	Chloroplast	1,037	114,618.80	6.21	−0.47	36.20	77.58
*RhlWRKY_19*	Chloroplast	235	26,337.87	7.65	−0.93	45.67	46.38
*RhlWRKY_20*	Nucleus	513	56,426.35	5.67	−0.91	58.40	60.45
*RhlWRKY_21*	Nucleus	327	36,311.32	5.53	−0.80	64.74	54.01
*RhlWRKY_22*	Nucleus	706	76,549.48	6.21	−0.77	50.02	58.73
*RhlWRKY_23*	Nucleus	314	34,865.89	6.13	−0.68	53.28	60.51
*RhlWRKY_24*	Nucleus	530	58,317.74	7.66	−0.82	56.51	56.64
*RhlWRKY_25*	Nucleus	353	37,583.77	5.45	−0.55	56.33	55.92
*RhlWRKY_26*	Nucleus	548	60,959.07	8.09	−0.96	55.80	46.82
*RhlWRKY_27*	Nucleus	341	38,169.53	5.59	−0.74	58.26	48.01
*RhlWRKY_28*	Nucleus	368	40,270.70	9.45	−0.51	43.52	72.64
*RhlWRKY_29*	Nucleus	382	41,956.37	5.90	−0.74	57.58	58.51
*RhlWRKY_30*	Nucleus	335	36,466.57	8.50	−0.82	46.45	64.06
*RhlWRKY_1*	Nucleus	669	72,676.20	5.83	−0.79	57.45	53.71
*RhlWRKY_32*	Nucleus	337	37,229.15	6.18	−0.71	52.26	65.64
*RhlWRKY_33*	Nucleus	265	29,339.31	5.39	−0.86	58.43	50.04
*RhlWRKY_34*	Nucleus	321	34,609.14	4.39	−0.57	71.41	64.74
*RhlWRKY_35*	Nucleus	324	36,260.44	6.70	−1.12	68.35	41.23
*RhlWRKY_36*	Nucleus	529	58,419.62	8.45	−1.00	61.29	42.59
*RhlWRKY_7*	Nucleus	333	36,835.36	8.79	−0.70	48.04	67.69
*RhlWRKY_38*	Nucleus	539	58,564.21	6.35	−0.68	44.20	61.48
*RhlWRKY_39*	Nucleus	596	64,701.54	6.19	−0.72	50.76	60.37
*RhlWRKY_40*	Nucleus	333	37,392.63	6.10	−0.95	75.25	45.92
*RhlWRKY41*	Nucleus	112	13,080.10	9.87	−0.83	46.54	59.91
*RhlWRKY_42*	Nucleus	584	63,627.68	6.88	−0.70	49.00	59.38
*RhlWRKY_43*	Nucleus	572	61,829.39	6.28	−0.73	44.64	62.71
*RhlWRKY_44*	Nucleus	492	53,384.50	9.07	−0.79	45.97	65.98
*RhlWRKY_45*	Nucleus	186	21,067.50	9.44	−0.91	36.35	51.34
*RhlWRKY_46*	Nucleus	321	34,644.23	4.39	−0.56	69.21	65.95
*RhlWRKY_47*	Nucleus	173	19,797.42	9.64	−0.86	49.91	55.14
*RhlWRKY_48*	Nucleus	210	23,878.47	4.54	−0.46	46.85	60.38
*RhlWRKY_49*	Nucleus	537	57,845.68	7.27	−0.80	62.56	54.90
*RhlWRKY_50*	Nucleus	280	31,264.80	5.44	−0.83	54.87	57.39
*RhlWRKY_51*	Nucleus	376	41,384.69	5.85	−0.76	60.19	56.06
*RhlWRKY_52*	Nucleus	489	53,513.58	6.40	−1.02	48.52	55.42
*RhlWRKY_53*	Nucleus	579	62,838.54	6.77	−0.72	47.18	53.71
*RhlWRKY_54*	Nucleus	347	39,043.18	5.46	−0.77	43.47	60.69
*RhlWRKY_55*	Nucleus	322	35,377.64	5.67	−0.62	50.87	67.52
*RhlWRKY_56*	Nucleus	113	13,129.42	9.76	−0.41	40.76	82.74
*RhlWRKY_57*	Nucleus	281	30,786.39	6.71	−0.62	70.46	40.42
*RhlWRKY_58*	Nucleus	281	30,929.59	7.07	−0.65	43.96	68.72
*RhlWRKY_59*	Nucleus	333	37,447.74	5.08	−0.62	46.62	62.67
*RhlWRKY_60*	Nucleus	329	36,314.42	8.14	−0.83	47.06	63.71
*RhlWRKY_61*	Nucleus	353	39,742.28	9.74	−0.76	45.65	65.44
*RhlWRKY_2*	Nucleus	289	32,291.19	5.23	−0.58	54.88	81.35
*RhlWRKY_63*	Nucleus	328	36,623.07	6.43	−0.65	54.85	62.50
*RhlWRKY_64*	Cytoplasm	159	18,242.76	9.26	−0.66	22.28	60.06
*RhlWRKY_65*	Chloroplast	256	28,908.49	8.19	−0.61	52.76	66.25

### Conservation domain and phylogenetic analysis of the WRKY gene family

A multiple sequence alignment was performed for the conserved domains of the *RhlWRKY* family, and a SeqLogo was generated (see Fig. S2). The majority of RhlWRKY transcription factors exhibit conservation in the N-terminal heptapeptide domain (*WRKYGQK*) and the C-terminal zinc finger (C2HH/C). These domains are similar to the amino acid variation patterns observed in WRKY proteins from rice and other species (Zhang & Wang, 2005). In addition to the conserved WRKYGQK sequence, three variants were identified: WKKYGEK in Group I (*RhlWRKY36*), WRKYGKK in Group II-c (*RhlWRKY64*), and WRKYGRK in Group II-d (*RhlWRKY65*).

**Figure 1:**
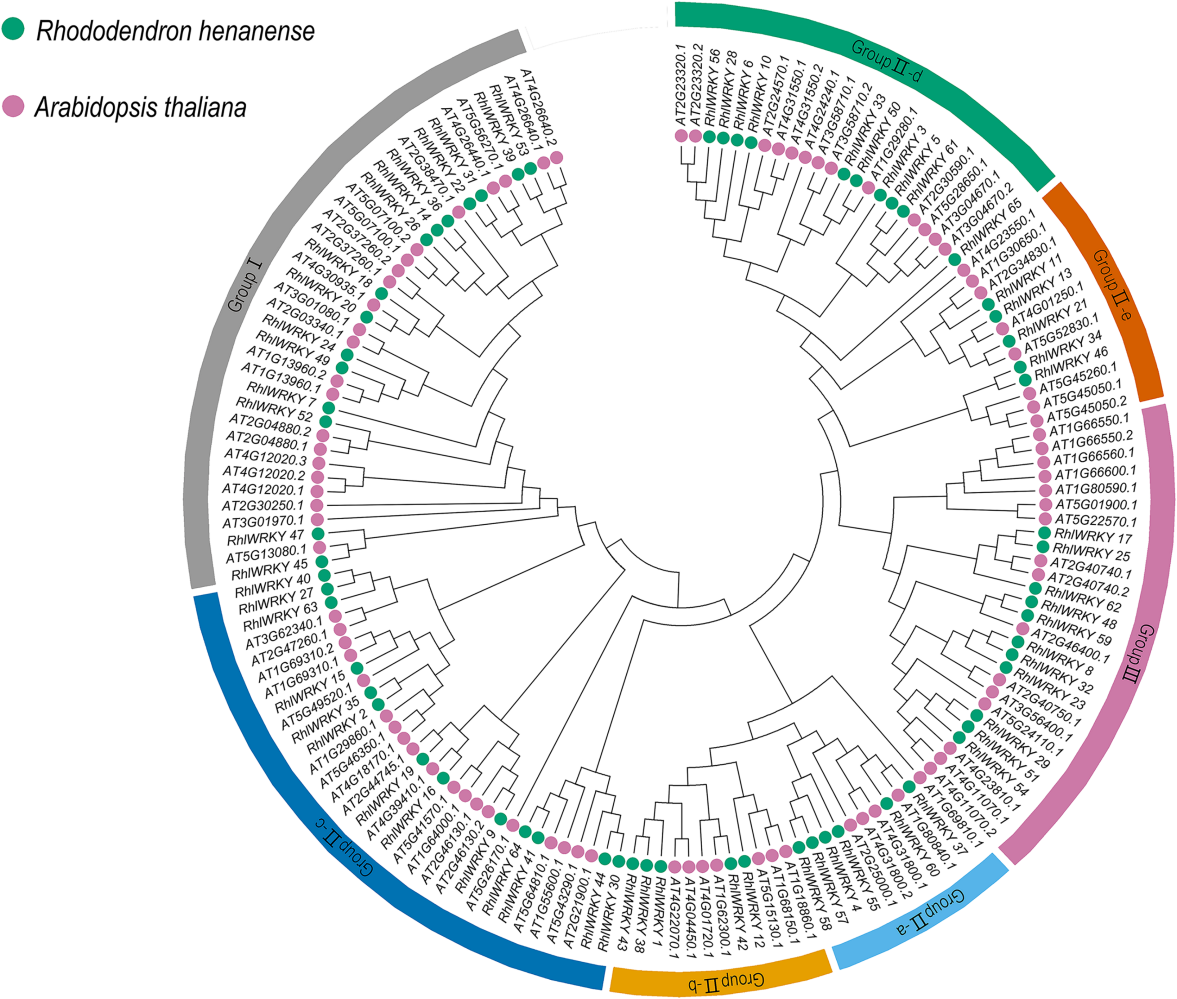
Phylogenic cluster of WRKY families in *R. henanense* subsp. *lingbaoense*. (65), *A. thaliana* (90).

A phylogenetic tree (see Fig. 1) was constructed by aggregating sequences of *WRKY* genes from *Rhl* (65) and Arabidopsis (90). WRKY transcription factors were categorized into three subgroups, with Group II further divided into five subclasses: Group II-a, -b, -c, -d, and -e. The three subgroups are in the following order, from largest to smallest: Group II, Group I, and Group III, with a total of 92, 37, and 27 *WRKY* genes, respectively.

### Gene structure and conserved domains of *RhlWRKY*

**Figure 2:**
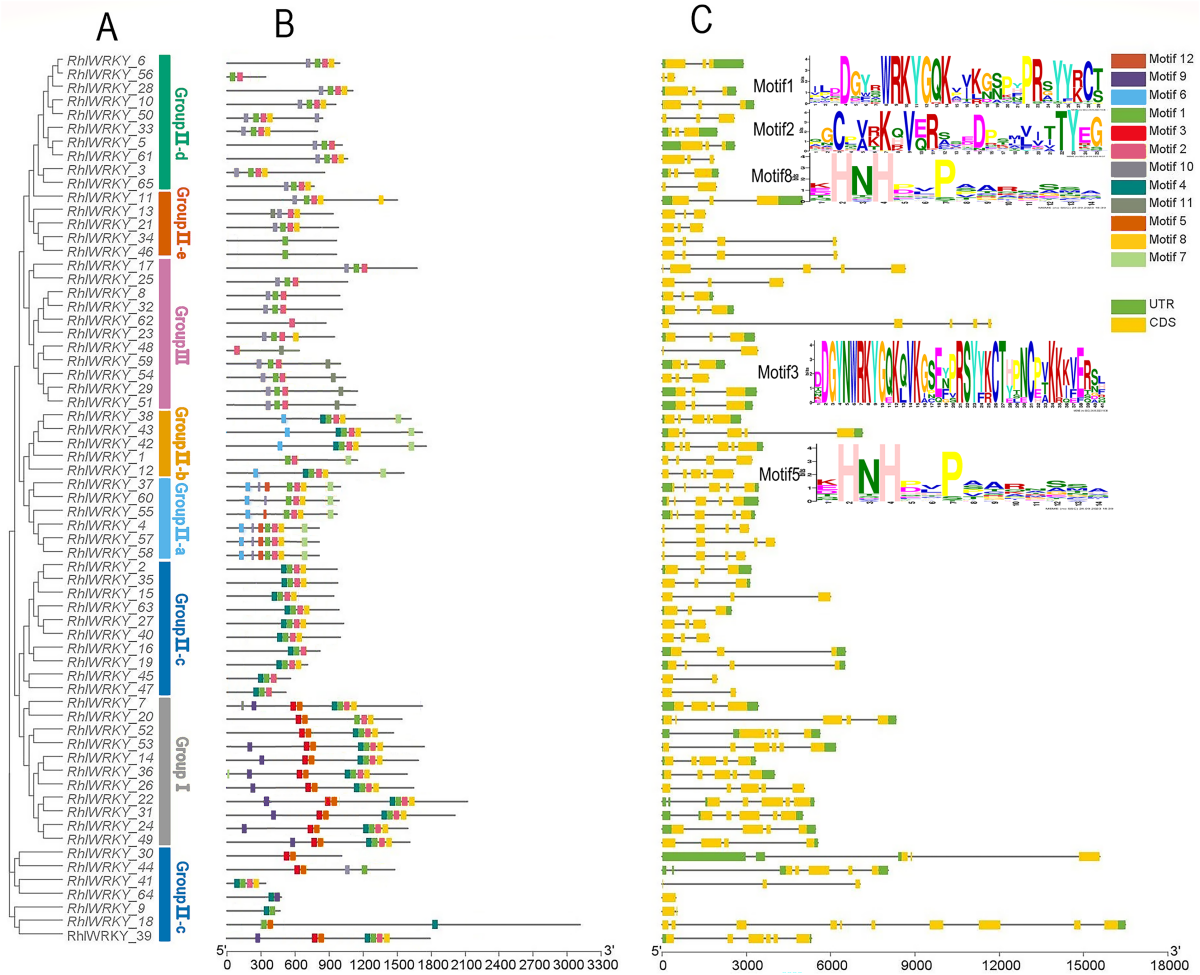
Phylogenetic relationships, conserved motifs and gene structure of *R. henanense subsp. lingbaoense*. (A) The phylogenetic tree prepared using sequences of 65 WRKY proteins from *R. henanense subsp. lingbaoense*. (B) The motif patterns of 65 WRKY Proteins. A total of 12 motifs are shown by the box in different colors. (C) The genetic map is shown. Yellow box, black line, and green box represent CDS, introns, and UTS, respectively.

Figure 2 shows that *RhlWRKY* genes contain 0–9 introns and 1–10 exons, and genes within the same subtribe exhibit similar exon-intron structures. This indicates that the 65 *RhlWRKY* genes have relatively conserved genetic structures. A total of 12 conserved motifs were identified within the RhlWRKY genes, and are designated as motifs 1–12. Motifs 1, 2, 8, and 4 are found in the majority of *Rhl* genes, indicating a high level of conservation in these four motifs. Among these, motif1 and motif3 contain the WRKYGQK conserved domain. Motif3, with the presence of CC (cysteine), and motif5, with HH/HC, together form the complete C2HH/C2HC zinc finger. Motif1 (containing C), motif2 (containing C), and motif8, or nearby sequences (containing HH), form a complete C2HH zinc finger. As shown in Fig. 2, nearly all genes contain conserved domains and zinc fingers. In Group I, there are two WRKY domains and two C2H2 zinc fingers. In Group II, there is one WRKY domain and one C2H2 structure, with Group II-a and -b containing a unique motif6 and Group II-d and -e mostly containing motif10, suggesting genes in these groups may have similar functions. In Group III, there is one WRKY domain and one C2HC zinc finger. Within the same subtribe, conserved motifs exhibit similarities, indicating that genes within the same subgroup likely share similar biological functions.

### Analysis of *cis*-acting elements

**Figure 3:**
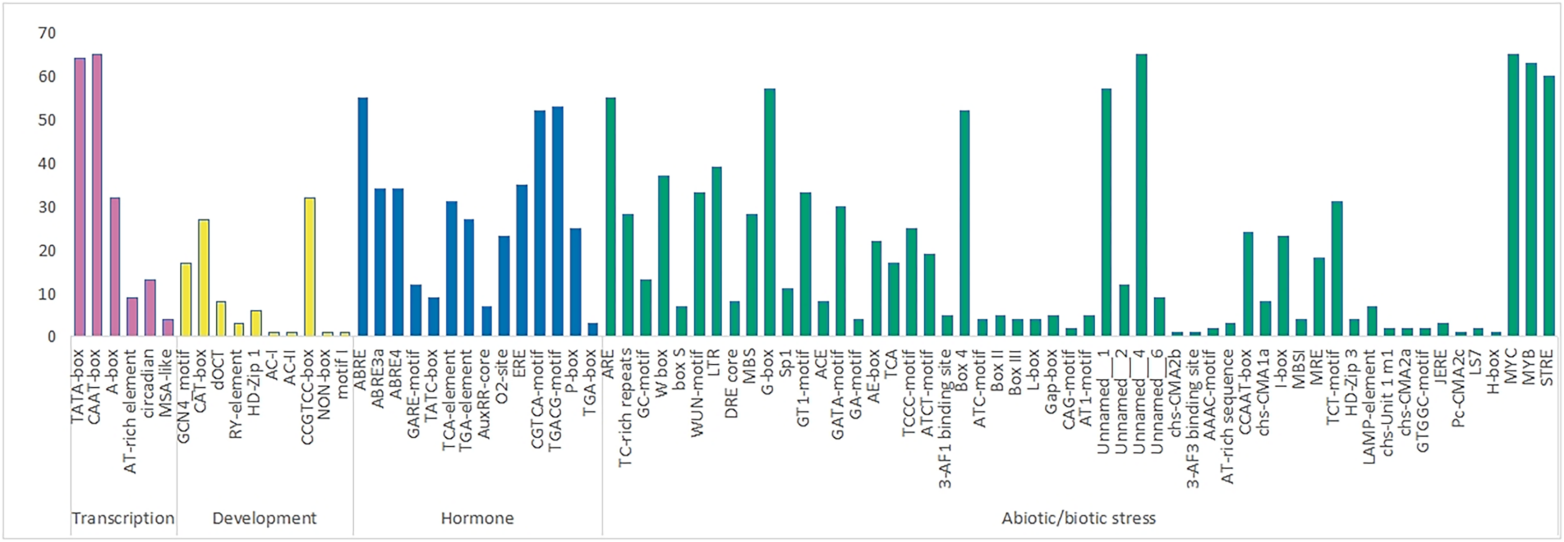
The number of various *cis* acting elements in the WRKY gene of *R.henanense* subsp. *lingbaoense*.

A total of 84 elements were identified in the upstream promoter regions of *RhlWRKY* (Fig. 3), and the results revealed the presence of various *cis*-elements in the flanking regions associated with stress, hormones, transcription and development *etc*. The majority of these elements fall into the stress response category, including elements associated with low-temperature, injury, and drought responses. Among the genes, 63 contain low-temperature response elements (MYB), 33 sequences exhibit wound response elements (WUM-motif), and all 65 genes contain drought response elements (MYC). Additionally, 28 genes have TC-rich repeat sequences, known to protect plants from damage in adverse conditions (Sun et al., 2016), and 18 genes feature light response elements (MRE). A total of 37 genes contain W-box elements, participating not only in wound and pathogen stress responses (Jiang et al., 2016) but also in self-regulation and cross-regulation with other genes (Chi et al., 2013). In terms of hormone response elements, 52 genes contain MeJA-induced response elements (CGTCA-motif), and 55 genes have abscisic acid response elements (ABRE). All 65 genes contain transport *cis*-acting elements (CAAT-box).

### Chromosome localization, collinearity analysis and Ka/Ks analysis

**Figure 4:**
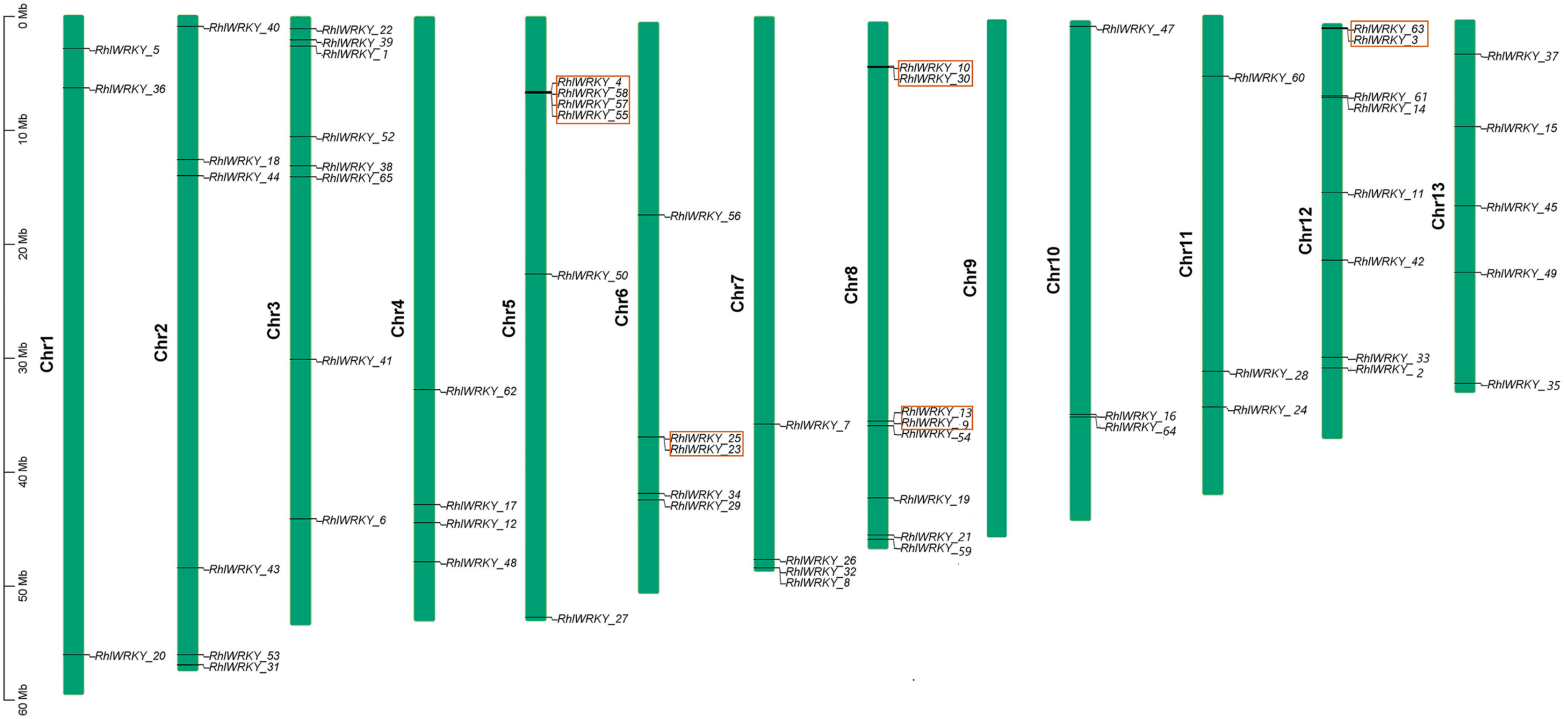
Chromosomal localization of WRKY Gene in *R.henanense* subsp. *lingbaoense* (Note: the red box shows tandem repeat gene pairs).

**Figure 5:**
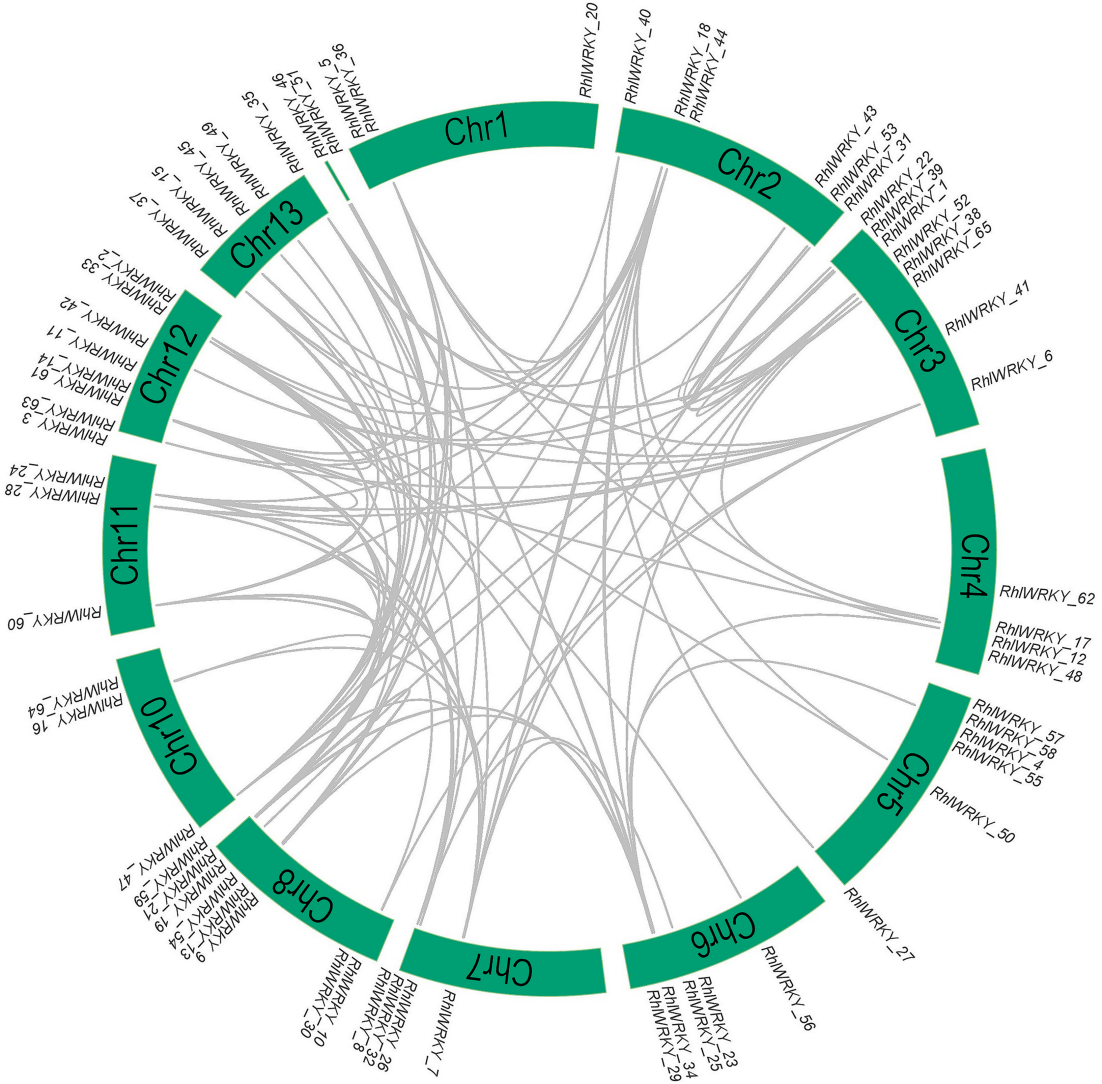
Collinearity analysis of WRKY genes in *R.henanense* subsp. *lingbaoense*.

The results of chromosomal location analysis (as shown in Fig. 4) indicate that out of the 65 *RhlWRKY* gene family members, only two genes, *RhlWRKY_46* and *RhlWRKY_51*, were not mapped to definitive chromosomal locations. It may be due to the fact that the scaffold could not be assigned to a chromosome. The remaining *RhlWRKY* family genes are distributed across 12 chromosomes, with the exception of chromosome 9, indicating a relatively small preference for the chromosomal distribution of *RhlWRKY* genes. The highest numbers of genes mapped to Chr3, Chr8, and Chr12, each with eight genes, while the fewest genes mapped to Chr1, Chr10, and Chr11, each with three genes. Two genes separated by five or fewer genes and located in a chromosomal segment less than 100 kb in length are considered tandem duplicate genes (Liu et al., 2020). Based on these criteria, a total of eight pairs of tandem duplicate sequences were identified (see Fig. 4, and Table S3), including four pairs on chromosome 5, two pairs on chromosome 8, and one pair on chromosome 6. Collinearity analysis revealed the presence of 97 segmental duplication relationships among the 65 *RhlWRKY* genes across the 12 chromosomes (see Fig. 5, and Table S3). To study evolutionary factors, Ka/Ks values were calculated for 105 duplicate gene pairs (see Table S3). A Ka/Ks ratio less than 1 indicates that gene pairs are in a state of negative selection or purifying selection, a Ka/Ks ratio greater than 1 indicates positive selection, and a Ka/Ks ratio equal to 1 suggests neutral selection (Wang et al., 2018). The homologous gene pair of *RhlWRKY_18* and *RhlWRKY_60* has a Ka/Ks ratio greater than 1, indicating positive selection, but the remaining 104 homologous pairs have Ka/Ks values less than 1, suggesting that the vast majority of *RhlWRKY* genes underwent purification due to environmental pressures.

### GO functional annotation analysis

**Figure 6:**
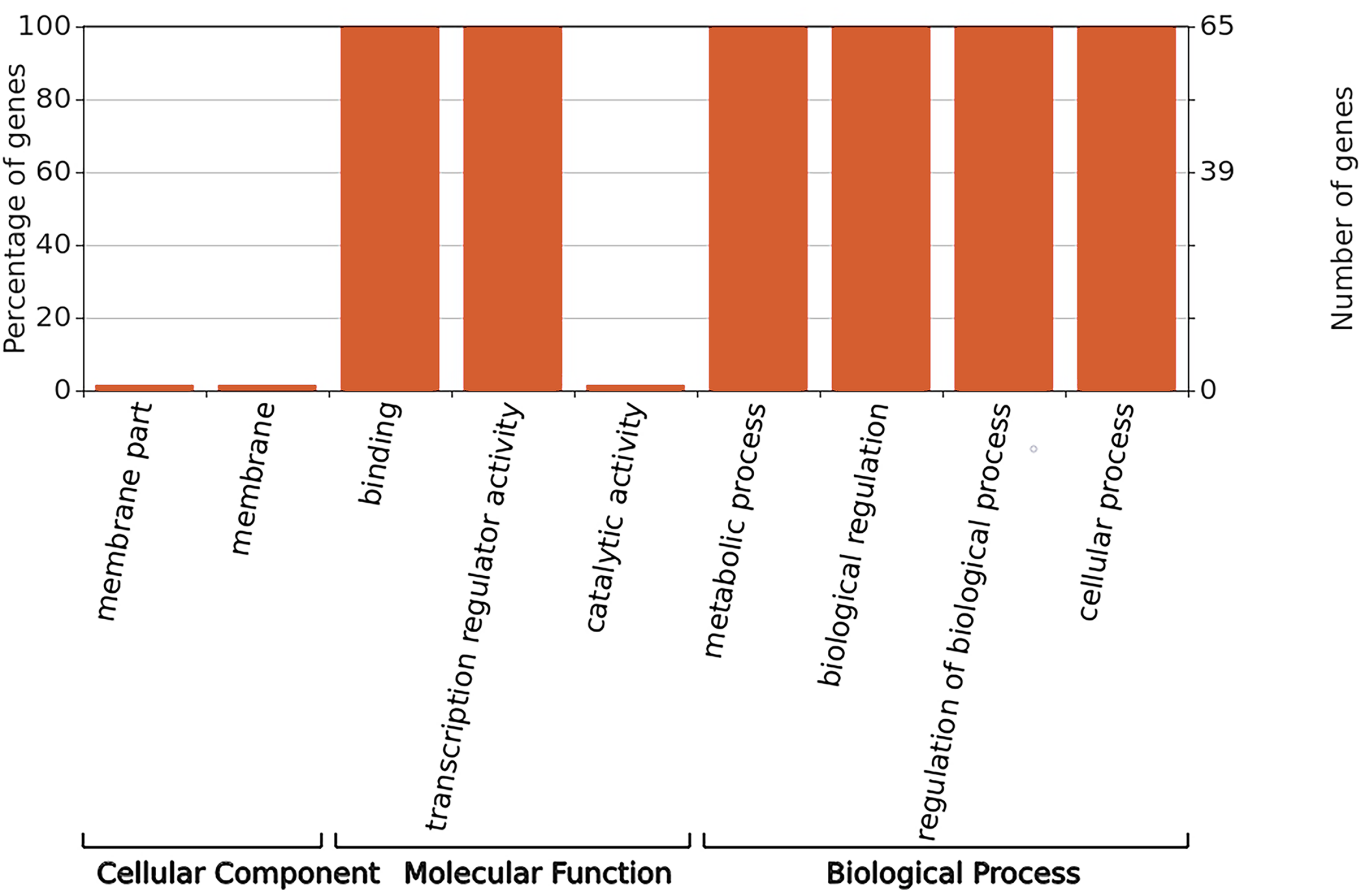
GO functional annotation of *RhlWRKY* genes in *R.henanense* subsp. *lingbaoense*.

GO annotation was used to classify gene functions into three categories: molecular functions, cellular components, and biological processes. As shown in Fig. 6, WRKY proteins are almost entirely absent in the cellular component category. Instead, these genes are primarily associated with molecular functions related to binding and transcription regulation activity. In terms of biological processes, WRKY proteins are involved in metabolic processes, biological regulation, regulation of biological processes, and cellular processes.

### Transcriptional abundance analysis of the *RhlWRKY* gene family

**Figure 7:**
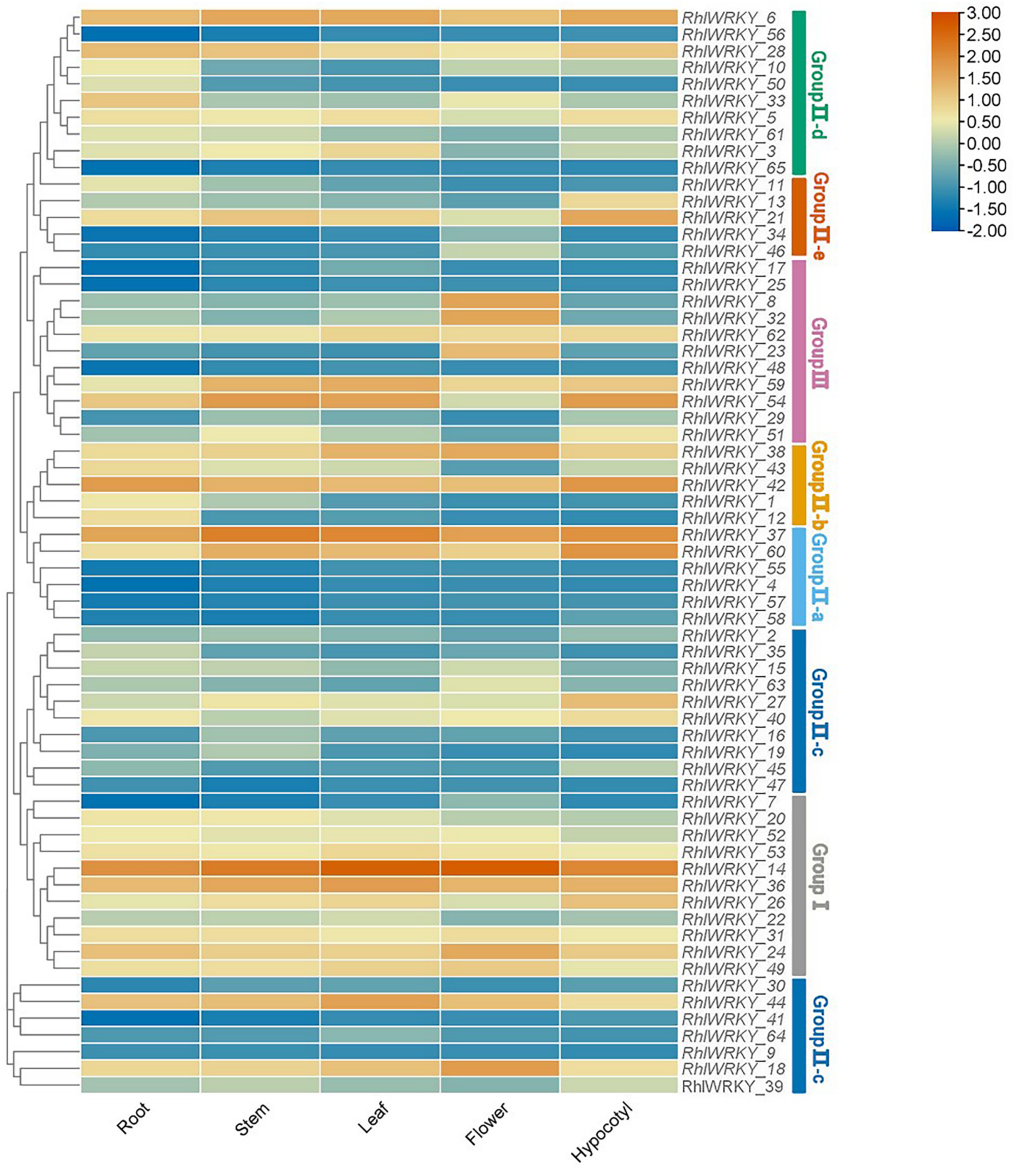
Tissue specific expression analysis of *RhlWRKY* family genes. The color code shown on the top of the figure represents different log2 values.

Transcriptional patterns were determined based on RNA-seq data from five different tissues of *Rhl*: roots, stems, leaves, flowers, and hypocotyls (see Fig. 7). R*hlWRKY_56* and *_65* were not detected in any of the five tissues, suggesting these might be pseudogenes. There were substantial differences in the expression profiles of various *RhlWRKY* gene family members across different tissues. Among the 43 *RhlWRKY* genes, 66.15% were expressed in all five tissues, with 24 of them showing relatively high expression levels (36.92%, FPKM > 4). Combining the *RhlWRKY* cluster analysis, it is evident that, in Group I, apart from *RhlWRKY_7*, the rest of the genes exhibited relatively high expression levels in all five tissues, with *RhlWRKY_14* exhibiting the highest expression level. In Group II, *RhlWRKY_5*, *_6*, *_18*, *_21*, *_37*, *_38*, *_42*, *_44*, and *_60* showed relatively high expression levels in all five tissues. *RhlWRKY_4*, *_9*, *_30*, *_34*, *_41*, *_57*, and *_64* exhibited lower expression levels or were undetectable in all five tissues. In Group III, *RhlWRKY_54*, *_59*, and *_62* showed relatively high expression levels in all five tissues, whereas *RhlWRKY_17*, *_25*, and *_48* had lower expression levels. As shown in Fig. 7, *RhlWRKY_14* is highly expressed in all tissues (with FPKM values of 236.62, 390.34, 360.22, 255.24, and 322.50 in roots, stems, leaves, flowers, and hypocotyls, respectively). Additionally, tissue-specific expression patterns were observed, with *RhlWRKY_37* showing specificity in stems, leaves, and hypocotyls. Genes with relatively high expression levels in stems included *RhlWRKY_6* and *_36*, and *RhlWRKY_60*, *_42*, *_21*, and *_6* exhibited higher expression levels in hypocotyls. Furthermore, *RhlWRKY_18* displayed specific expression in flowers, and *RhlWRKY_42* and *_37* showed tissue-specific expression in roots. These genes likely play key roles in their respective tissues.

### Analysis of expression patterns under abiotic stress

**Figure 8:**
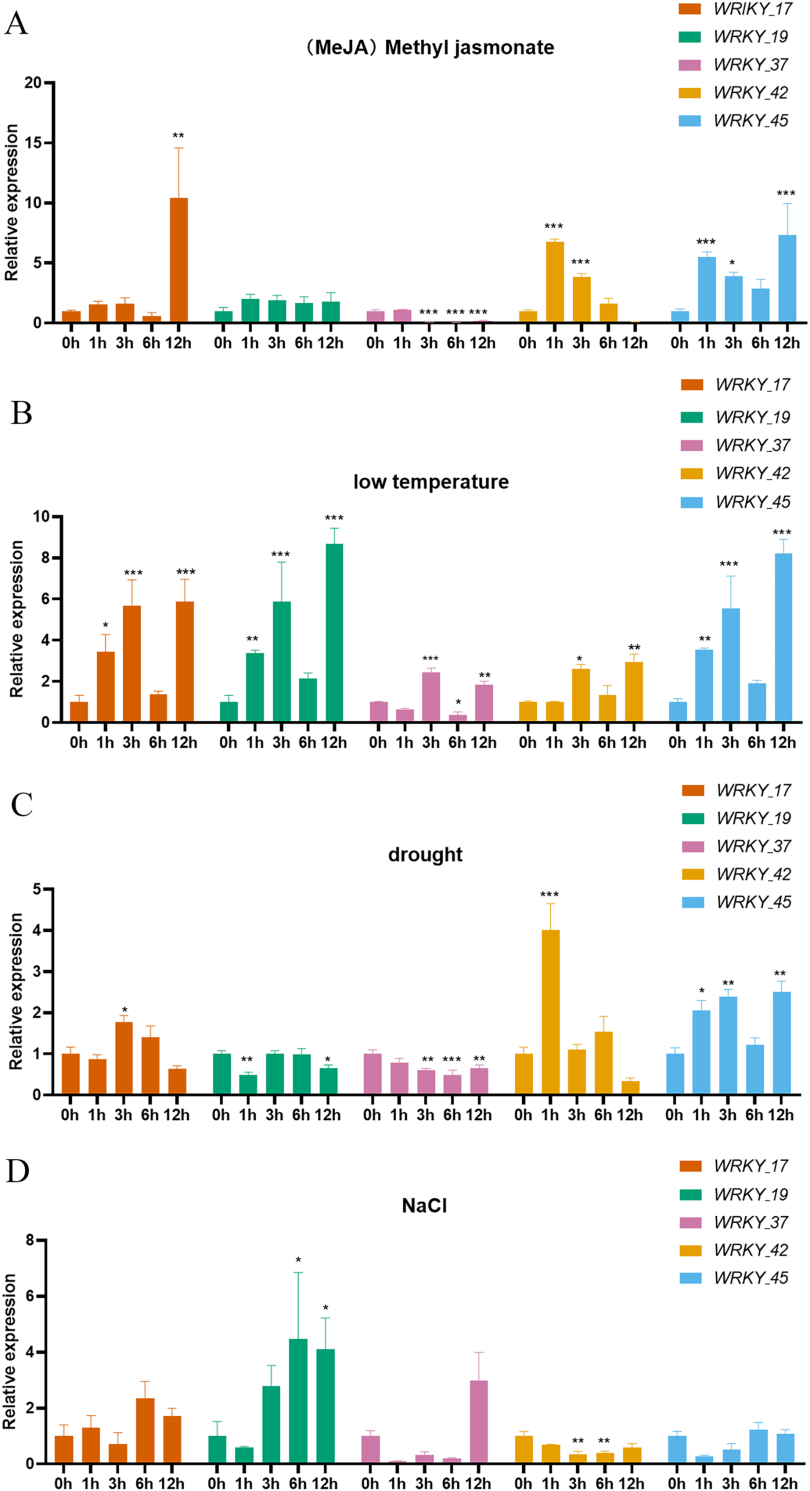
Expression of 5 WRKY genes under different abiotic factor stress in *R. henanense* subsp. *lingbaoense*. (A) MeJA (B) 4 °C (C) PEG (D) NaCl. Statistical analysis is performed using an one-way analysis of variance (Note *P < 0.05 **P < 0.01 ***P < 0.001).

Expression analysis was performed on five genes belonging to three subtribes of the *WRKY* gene family. The five selected genes (*RhlWRKY_17*,*_19*,*_37*,*_42,_45*) show relatively high expression levels in the selected tissues and were orthologous to *Arabidopsis* (Fig. 8). Under MeJA treatment, *WRKY_17* exhibited significant upregulation at 12 h, and *WRKY_37* showed significant down-regulation at 3, 6, and 12 h. *WRKY_42* showed significant up-regulation at 1 and 3 h, and *WRKY_45* had significant up-regulation at 1 and 12 h with respect to 0 h. Under low-temperature stress, *WRKY_17* showed significant up-regulation at 1 and 3 h, *WRKY_19* was up-regulated at 3 and 12 h, *WRKY_37* was up-regulated at 3 h and further at 12 h, *WRKY_42* showed increased expression at 12 h, and *WRKY_45* showed higher expression at 3 and 12 h with respect to 0 h. In response to drought stress, *WRKY_17* exhibited upregulation at 3 h, *WRKY_19* showed significant downregulation at 1 h, and *WRKY_37* showed downregulation at 3 and 12 h, with a more significant decrease at 6 h. Additionally, *WRKY_42* showed significant upregulation at 1 h, and *WRKY_45* had significant upregulation at 3 h and 12 h with respect to 0 h. Under high-salinity stress, *WRKY_19* had upregulation at 6 h and 12 h, while *WRKY_42* showed a more significant down-regulation at 3 h and 6 h with respect to 0 h. *WRKY_17* exhibited a 10-fold upregulation at 12 h under MeJA treatment with respect to 0 h, suggesting this is a major gene that responds to MeJA treatment. *WRKY_19* showed an 8.7-fold upregulation at 12 h under low-temperature treatment with respect to 0 h, indicating it as the major gene to respond to low-temperature stress. *WRKY_42* showed a 4-fold upregulation at 1 h under drought treatment with respect to 0 h, indicating it as a major gene involved in drought response. Interestingly, *WRKY_42* exhibited relatively high expression levels across various tissues (roots, stems, leaves, flowers, and hypocotyls). *WRKY_19* showed a 4.5-fold upregulation at 6 h under high-salinity treatment with respect to 0 h, indicating that this gene likely acts to counter high-salinity stress.

## Discussion

WRKY transcription factors activate or inhibit the expression of downstream genes through self-regulation or cross-regulation, participating in various biological processes of plant growth, development, and stress responses (Sun et al., 2020). The *WRKY* gene family has been studied in various plants such as *Arabidopsis* (Eulgem et al., 2000), sweet orange (Silva, Ito & Souza, 2017), sesame (Li et al., 2017), cassav ((Wei et al., 2016), maize (Hu et al., 2021), sunflower (Li et al., 2020), and osmanthus (Ding et al., 2020). However, this identification and characterization of WRKY transcription factors in *Rhl* represents a novel contribution to the field. In this study, a total of 65 *RhlWRKY* genes were identified, and subcellular localization analysis revealed that most RhlWRKY proteins are predicted to be located in the cell nucleus, consistent with the fundamental characteristic of transcription factors being nuclear proteins (Fu et al., 2019) (Table 1). Based on phylogenetic analysis (Fig. 2), the *RhlWRKY* gene family can be divided into three subtribes, Group II, I, and III, in order from largest to smallest. The distribution is similar to that in *Arabidopsis*. Ten pairs of homologous genes were found between *Arabidopsis* and *Rhl*, suggesting these genes may have similar functions (Zhu, 2021). For instance, overexpression of *Arabidopsis AtWRKY75* accelerates leaf aging (Zhang et al., 2021), and overexpression of its orthologous gene *RhlWRKY_45* may also accelerate leaf aging. Multiple sequence alignment (Fig. S2) showed that most RhlWRKY proteins share a conserved heptapeptide sequence (WRKYGQK) at their N-terminus and a shared zinc-finger at their C-terminus. However, other variants, such as WKKY-GEK, WRKYGKK, and WRKYGRK, were also found in this analysis. This variation has been observed for other *WRKY* genes in *Arabidopsis*, rice, apple, grape, and potato (Eulgem et al., 2000; Ross, Yue & Shen, 2007; Meng et al., 2016; Guo et al., 2014; Zhang et al., 2017). Perhaps these different variants will bind to distinct elements, not just W-box elements (Eulgem et al., 2000). Eight WRKY proteins lacking a complete zinc-finger motif and four proteins with incomplete WRKYGQK sequences were also identified as members of the *RhlWRKY* gene family. Gain and loss of structural domains may partially explain the expansion of the *WRKY* gene family in *Rhl*.

Conserved motif analysis (see Fig. 2) revealed that the majority of *WRKY* genes possess motif1, motif2, motif8, and motif4, suggesting these may be important features for identifying *WRKY* genes. Similar conserved motifs exist within the same subtribe, but significant differences can be observed between different subgroups, consistent with the diverse functions of the *WRKY* gene family (Zheng et al., 2022). Genetic structure analysis (Fig. 2) showed that homologous genes have a generally similar structure, but there are also irregularities. For example, the two genes in the homologous gene pair of *RhlWRKY_17* and *RhlWRKY_25* vary in the number of introns, with four and two, respectively. This discrepancy might be due to the loss, gain, or alteration of introns during the evolution of the *WRKY* gene family (Rogozin et al., 2005). Genes with intron loss can accelerate evolution through duplication processes (Lecharny et al., 2003).

The analysis of *cis*-acting elements (Fig. 4) revealed that most are related to stress responses, including elements responsive to MeJA, low-temperature, and drought. Chromosomal mapping (Fig. 4) indicates that 28.6% of *RhlWRKY* genes are clustered, which can facilitate sequence exchange between genes (Shui et al., 2023). As shown in Fig. 4, there are eight pairs originating from tandem duplication events, and 97 pairs of segmental duplication events were found to have a collinearity relationship. This suggests that segmental duplication played a crucial role in the expansion of this family (Li et al., 2022). Previous research has shown that gene duplication largely explains the emergence of new genes (Yin et al., 2013). Gene duplication can lead to sub-functionalization. For example, the function of wheat *WRKY* gene family members expanded through tandem and whole-genome duplications (Hassan et al., 2019). Analyzing the Ka and Ks substitution rates in duplicate genes provides insight into the evolution of important genes (Hanada, Shiu & Li, 2007). In 104 out of 105 duplicated pairs, the Ka/Ks ratio is <1 (see Table S3), indicating that these gene pairs have undergone strong purifying selection. Purifying selection typically removes harmful alleles selectively over time (Biswas & Akey, 2006), suggesting that the *WRKY* gene family likely plays a significant role in the development and survival of *Rhl*, making the preservation and propagation of all its members necessary. Only one pair of homologous genes, *RhlWRKY_18* and *RhlWRKY_60*, has a Ka/Ks ratio >1, indicating positive selection and carrying important implications for species evolution (Li et al., 2023). According to the results of GO functional annotation (see Fig. 6), RhlWRKY transcription factors perform various molecular functions and regulate various cellular metabolic processes.

Transcriptome expression profile analysis reveals that three genes, *RhlWRKY_4*, *_56*, and *_65*, show minimal expression levels in the roots, stems, leaves, flowers, and hypocotyls of *Rhl*. In five tissues, 24 *RhlWRKY* members exhibit relatively high expression levels in these five tissues, while several of the remaining *RhlWRKYs* exhibit significant tissue-specific expression patterns. This suggests that *RhlWRKYs* may have various roles in regulating growth, development, and secondary metabolism in *Rhl*. Interestingly, *RhlWRKY_8*, *_23*, and *_32* have higher expression levels in flowers. These three genes have orthologous genes in *Catharanthus roseus* that regulate the biosynthesis of terpenoid indole alkaloids, such as *CrWRKY1* (*At3G56400* and *At2G40750* orthologous genes) (Schluttenhofer et al., 2014). Therefore, *RhlWRKY_8*, *_23*, and *_32* may be involved in the regulation of alkaloid secondary metabolism in *Rhl*. The essential roles of WRKY TFs in plant growth, development, and stress resistance have been extensively studied, particularly in *Arabidopsis*, where many *WRKY* genes have been functionally characterized. Thus, identifying the closest Arabidopsis homologs for individual *RhlWRKY* genes can provide hints about their potential functions. For example, *At5G07100* regulates heat shock proteins and heat-induced ethylene-dependent responses (Li, Fu & Chen, 2011), so its orthologous gene *RhlWRKY_26* may have a similar function. Both *At4G23810* and *At3G56400*, which belong to Group III, play important roles in leaf aging (Chen et al., 2010) *RhlWRKY_54* and *_59* also belong to Group III and are highly expressed in leaves. The orthologous counterpart of *RhlWRKY_15* is *AT1G69310* in Arabidopsis, which can enhance drought tolerance by increasing abscisic acid levels (Srivastava et al., 2018). *AT4G31800* and *AT2G25000* enhance plant sensitivity to salt and osmotic stress, suggesting potential function for the orthologous *RhlWRKY_60* (Chen et al., 2010). *RhlWRKY_60* may also have a similar function, given its high expression levels in stems and hypocotyls. *AT4G31550* and *AT2G24570* act to defend against both biotic and abiotic stress (Liu et al., 2011). Their orthologous gene, *RhlWRKY_10*, may have similar functions, especially given its tissue-specific expression in roots.

The expression analysis (Fig. 8) indicates that *WRKY_17* is the major gene whose expression changes in response to MeJA treatment, *WRKY_19* is the major gene with changes in response to low-temperature and high-salinity treatments, and *WRKY_42* is the major gene that changes in response to drought treatment. *RhlWRKY_37* is an orthologous gene to *Arabidopsis AtWRKY40*, which is significantly expressed in response to drought stress, indicating a drought resistance function (Che et al., 2018). Interestingly, as shown in Fig. 8, the expression of *RhlWRKY_37* is significantly downregulated after 3 h of drought treatment, which is opposite to the behavior of the *AtWRKY40* gene. This difference might be due to variations in the environmental preferences of the two plants, as *Rhl* prefers partial shade to intense sunlight (Han et al., 2008), whereas *Arabidopsis* typically grows in dry outdoor soils (Zhao, 2011). Additionally, *RhlWRKY_37* has very high expression levels in roots, stems, leaves, flowers, and hypocotyls. *AtWRKY6* and *RhlWRKY_42* are a homologous gene pair, and *AtWRKY6* regulates seed germination. When its expression is low, it reduces the sensitivity of seeds to ABA, leading to faster seed germination, and with higher expression, germination is slower (Li et al., 2017). Thus, in addition to response to drought stress, the *RhlWRKY_42* gene may also regulate seed germination. *RhlWRKY_42* also exhibits high expression in roots, stems, leaves, flowers, and hypocotyls, making it a candidate gene for further research on the function of *RhlWRKY* genes.

## Conclusion

This study identified 65 *WRKY* genes in the genome of *Rhl* and analyzed their gene characteristics and expression patterns in different tissues and under various stress conditions. The results showed that *RhlWRKY* genes can respond to various hormones and abiotic stresses. Specifically, *WRKY_42* and *WRKY_17* were identified as genes changing in response to drought and MeJA treatment, respectively, and *WRKY_19* showed changes in expression in response to low-temperature and high-salinity conditions. These findings lay the foundation for further work to identify and characterize *WRKY* genes in Rhododendron. Additionally, through phylogenetic comparisons, the study made functional predictions for some genes and identified valuable candidate genes for *RhlbZIP* gene molecular mechanism, providing a basis for further functional research and feature analysis. This study laid a solid foundation for further analysis of, function and characteristics.

## Supplemental Information

10.7717/peerj.17435/supp-1 Supplemental Information 1 Sample randomness distribution curve.

10.7717/peerj.17435/supp-2 Supplemental Information 2 Conserved domain SeqLogo of the WRKY family of *R.henanense* subsp.*lingbaoense*.

10.7717/peerj.17435/supp-3 Supplemental Information 3 Supplemental Tables. Values of Ks, Ka, and Ka/Ks for Duplicate Gene Pairs

10.7717/peerj.17435/supp-4 Supplemental Information 4 WRKY genes expression raw data.

10.7717/peerj.17435/supp-5 Supplemental Information 5 MIQE checklist and detailed answer.

10.7717/peerj.17435/supp-6 Supplemental Information 6 Raw data exported from the CFX96 Real time PCR Detection System (Bio Rad, USA) for data analyses and preparation for Fig. 8 for the different stress treatments time of 0,1,3,6,12h.

10.7717/peerj.17435/supp-7 Supplemental Information 7 Raw data exported from the DNBSEQ-T7 (Wuhan Feisha Bioinformation Co., Ltd) for data analyses and preparation for Fig. 7 and Table S1 for the shoot, root, leaf, flower, and hypocotyl of Rhl.

## Additional Information and Declarations

### Data Availability

The following information was supplied regarding data availability: The RNA-seq data is available at NCBI: SAMN37367964–37367966 (root); SAMN37367967–37367969 (stem); SAMN37367970–37367972 (leaf); SAMN37367973–37367975 (hypocotyl); SAMN37367976–37367978 (flower).

## References

[ref-1] Biswas S, Akey JM (2006). Genomic insights into positive selection. Trends in Genetics.

[ref-2] Bu HH, Wang XQ, Ren ZQ, Xiao JH, Zhang N, Yang HZ (2020). Research progress on plant WRKY transcription factors family genes. Journal of Shanxi Agricultural Sciences.

[ref-3] Chang GD (2011). Research on chemical components and biological activities of Rhododendron micranthum Turcz.

[ref-4] Che YM, Sun YJ, Lu SC, Zhao FG, Hou LX, Liu X (2018). *AtWRKY40* functions in drought stress response in *Arabidopsis thaliana*. Plant Physiology Journal.

[ref-5] Chen H, Lai ZB, Shi JW, Xiao Y, Chen ZX, Xu XP (2010). Roles of *Arabidopsis WRKY18*, *WRKY40* and *WRKY60* transcription factors in plant responses to abscisic acid and abiotic stress. BMC Plant Biology.

[ref-71] Chen L, Song Y, Li SJ, Zhang L, Zou C, Yu D (2012). The role of WRKY transcription factors in plant abiotic stresses. Biochimica et Biophysica Acta (BBA)–Gene Regulatory Mechanisms.

[ref-6] Chi YJ, Yang Y, Zhou Y, Zhou J, Fan BF, Yu JQ, Chen ZX (2013). Protein-protein interactions in the regulation of WRKY transcription factors. Molecular Plant.

[ref-7] Conesa A, Götz S, García-Gómez JM, Terol J, Talón M, Robles M (2005). Blast2GO: a universal tool for annotation, visualization and analysis in functional genomics research. Bioinformatics.

[ref-8] Ding WJ, Ouyang QX, Li YL, Shi TT, Li L, Yang XL, Ji KS, Wang LG, Yue YZ (2020). Genome-wide investigation of WRKY transcription factors in sweet osmanthus and their potential regulation role in aroma synthesis. Tree Physiology.

[ref-9] Eulgem T, Rushton PJ, Robatzek S, Somssich IE (2000). The WRKY superfamily of plant transcription factors. Trends in Plant Science.

[ref-10] Fu MC, Hao Li, Chen YZ, Wang LG, Liu ZJ (2019). Genome-wide investigation of WRKY transcription factors in *Gossypium barbadense* and their expression patterns in response to *Verticillium dahliae* infection. Journal of Plant Genetic Resources.

[ref-11] Guo C, Guo RG, Xu XZ, Gao M, Li XQ, Song JY, Zheng Y, Wang XP (2014). Evolution and expression analysis of the grape (*Vitis vinifera* L.) WRKY gene family. Journal of Experimental Botany.

[ref-12] Han JW, Zhang Y, Yuan ZL, Ye YZ (2008). Study on the introduction and development of wild *Rhododendron lingbaoense* in Xiaoqinling National Nature Reserve of Henan. Journal of Anhui Agricultural Sciences.

[ref-13] Hanada K, Shiu S-H, Li W-H (2007). The nonsynonymous/synonymous substitution rate ratio versus the radical/conservative replacement rate ratio in the evolution of mammalian genes. Molecular Biology and Evolution.

[ref-14] Hassan S, Lethin J, Blomberg R, Mousavi H, Aronsson H (2019). In silico based screening of WRKY genes for identifying functional genes regulated by WRKY under salt stress. Computational Biology and Chemistry.

[ref-15] Hu W, Ren Q, Chen Y, Xu G, Qian Y (2021). Genome-wide identification and analysis of WRKY gene family in maize provide insights into regulatory network in response to abiotic stresses. BMC Plant Biology.

[ref-16] Huang X, Ding F, Peng HX, Pan JC, He XH, Xu JZ, Li L (2019). Research progress on family of plant WRKY transcription factors. Biotechnology Bulletin.

[ref-17] Jiang YZ, Duan YJ, Yin J, Ye SL, Zhu JR, Zhang FQ, Lu WW, Fan D, Kem L (2015). Genome-wide identification and characterization of the WRKY transcription factor family and analysis of their expression in response to biotic and abiotic stresses. Genome.

[ref-18] Jiang M, Liu Q, Liu ZN, Li JZ, He CM (2016). Over-expression of a WRKY transcription factor gene BoWRKY6 enhances resistance to downy mildew in transgenic broccoli plants. Australasian Plant Pathology.

[ref-19] Ku SK, Zhou W, Lee W, Han MS, Na MK, Bae JS (2015). Anti-inflammatory effects of hyperoside in human endothelial cells and in mice. Inflammation.

[ref-20] Lecharny A, Boudet N, Gy I, Aubourg S, Kreis M (2003). Introns in, introns out in plant gene families: a genomic approach of the dynamics of gene structure. Journal of Structural and Functional.

[ref-21] Lescot M, Déhais P, Thijs G, Marchal K, Moreau Y, Van de Peer Y, Rouzé P, Rombautset S (2002). PlantCARE, a database of plant *cis*-acting regulatory elements and a portal to tools for in silico analysis of promoter sequences. Nucleic Acids Research.

[ref-22] Li S, Fu Q, Chen L (2011). *Arabidopsis thaliana WRKY25*, *WRKY26*, and *WRKY33* coordinate induction of plant thermotolerance. Planta.

[ref-23] Li LQ, Huang LP, Gang P, Liu L, Wang XY, Lu LM (2017). Identifying the genes regulated by *AtWRKY6* using comparative transcript and proteomic analysis under phosphorus deficiency. International Journal of Molecular Sciences.

[ref-24] Li J, Islam F, Huang Q, Wang J, Zhou W, Xu L, Yang C (2020). Genome-wide characterization of WRKY gene family in Helianthus annuus L. and their expression profiles under biotic and abiotic stresses. PLOS ONE.

[ref-25] Li YH, Liang YJ, Chang SJ, Guo MX, Zhang YZ, Chen Y (2023). Genome-wide identification and expression features of AP2 gene family in *Hypericum perforatum*. Journal of Agricultural Biotechnology.

[ref-26] Li TQ, Liu XF, Li Z, Li ZH, Ma H, Wan YM, Liu XX, Fu LY (2018). Study on reproductive biology of *Rhododendron longipedicellatum*: a newly discovered and special threatened plant surviving in limestone habitat in southeast Yunnan, China. Frontiers in Plant Science.

[ref-27] Li DH, Liu P, Yu JY, Wang LH, Dossa K, Zhang YX, Zhou R, Wei X, Zhang XR (2017). Genome-wide analysis of WRKY gene family in the sesame genome and identification of the WRKY genes involved in responses to abiotic stresses. BMC Plant Biology.

[ref-28] Li ML, Su JJ, Yang YL, Qin JH, Li XX, Yang DL, Ma Q, Wang CX (2022). Identification of *COI* family genes and their expression in *Gossypium hirsutum* L. under drought and salt stress. Journal of Agricultural Science and Technology.

[ref-29] Liang JY, You CX, Guo SS, Guo SS, Zhang WJ, Li Y, Geng ZF, Wang CF, Du SS, Deng ZW, Zhang J (2016). Chemical constituents of the essential oil extracted from Rhododendron thymifolium and their insecticidal activities against Liposcelis bostrychophila or Tribolium castaneum. Industrial Crops and Products.

[ref-30] Liu Z, Coulter JA, Li YM, Zhang XJ, Meng JG, Zhang JL, Liu YH (2020). Genome-wide identification and analysis of the Q-type C2H2 gene family in potato (*Solanum tuberosum* L.). International Journal of Biological Macromolecules.

[ref-31] Liu HY, Yang WL, Liu DC, Han YP, Zhang AM, Li SH (2011). Ectopic expression of a grapevine transcription factor VvWRKY11 contributes to osmotic stress tolerance in *Arabidopsis*. Molecular Biology Reports.

[ref-32] Matsui A, Ishida J, Morosawa T, Mochizuki Y, Kaminuma E, Endo TA, Okamoto M, Nambara E, Nakajima M, Kawashima M, Satou M, Kim JM, Kobayashi N, Toyoda T, Shinozaki K, Seki M (2008). Arabidopsis transcriptome analysis under drought, cold, high-salinity and ABA treatment conditions using a tiling array. Plant and Cell Physiology.

[ref-33] Meng D, Li Y, Bai Y, Li M, Cheng L (2016). Genome-wide identification and characterization of WRKY transcriptional factor family in apple and analysis of their responses to waterlogging and drought stress. Plant Physiology and Biochemistry.

[ref-34] Peng T, Wang YQ, Chen MM, Feng LP, Bao MZ, Zhang JW (2019). Cloning and expression analysis of *PmWRKY40* gene in prunus mume. Journal of Huazhong Agricultural University.

[ref-35] Pillai SE, Kumar C, Patel HK, Sonti RV (2018). Overexpression of a cell wall damage induced transcription factor, OsWRKY42, leads to enhanced callose deposition and tolerance to salt stress but does not enhance tolerance to bacterial infection. BMC Plant Biology.

[ref-36] Rogozin IB, Sverdlov AV, Babenko VN, Koonin EV (2005). Analysis of evolution of exon-intron structure of eukaryotic genes. Briefings in Bioinformatics.

[ref-37] Ross CA, Yue L, Shen QJ (2007). The WRKY gene family in Rice (Oryza sativa). Journal of Integrative Plant Biology.

[ref-38] Schluttenhofer C, Pattanaik S, Patra B, Yuan L (2014). Analyses of *Catharanthus roseus* and *Arabidopsis thaliana* WRKY transcription factors reveal involvement in jasmonate signaling. BMC Genomics.

[ref-39] Shui DJ, Sun J, Xiong ZL, Xu HW, Zhang SM, Shi JL (2023). Comparative identification of WRKY transcription factors and bacterial stress response in tomato. Fujian Journal of Agricultural Sciences.

[ref-40] Silva EG, Ito TM, Souza GH (2017). In silico genome-wide identification and phylogenetic analysis of the WRKY transcription factor family in sweet orange (*Citrus sinensis*). Australian Journal of Crop Science.

[ref-41] Srivastava R, Kumar S, Kobayashi Y, Kusunoki K, Tripathi P, Kobayashi Y, Koyama H, Sahoo L (2018). Comparative genome-wide analysis of WRKY transcription factors in two Asian legume crops: Adzuki bean and Mung bean. Scientific Reports.

[ref-42] Sun JL, Dong YM, Wang CQ, Xiao SH, Wang X, Li LB, Jiao ZG (2016). Identification and characterization of NRT3 genes from cucumber. Chinese Agricultural Science Bulletin.

[ref-43] Sun C, Wang CX, Liang Y, Li CM, Zhang ZY, Jiao XJ, Han CM (2020). Research progress of plant WRKY transcription factors. Journal of Shandong Forestry Science and Technology.

[ref-44] Vives-Peris V, Marmaneu D, Gomez-Cadenas A, Perez-Clemente RM (2018). Characterization of Citrus WRKY transcription factors and their responses to phytohormones and abiotic stresses. Biologia Plantarum.

[ref-45] Wang SL, Chu ZH, Jia R, Fei D, Shen XL, Li Y, Ding XH (2018). SlMYB12 regulates flavonol synthesis in three different cherry tomato varieties. Scientific Reports.

[ref-46] Wang QQ, Liu F, Chen XS, Ma XJ, Zeng HQ, Yang ZM (2010). Transcriptome profiling of early developing cotton fiber by deep-sequencing reveals significantly differential expression of genes in a fuzzless/lintless mutant. Genomics.

[ref-48] Wei YX, Shi HT, Xia ZQ, Tie WW, Ding ZH, Yan Y, Wang WQ, Hu W, Li K (2016). Genome-wide identification and expression analysis of the WRKY gene family in cassava. Frontiers in Plant Science.

[ref-49] Wei X, Wang HT, Wei HL, Fu XK, Ma L, Lu JH, Wang SF, Yu SX (2020). Cloning and drought resistance analysis of GhWRKY33 in upland cotton. Scientia Agricultura Sinica.

[ref-50] Yin G, Xu H, Xiao SY, Qin YJ, Li YX, Yan YM, Hu YK (2013). The large soybean (Glycine max) WRKY TF family expanded by segmental duplication events and subsequent divergent selection among subgroups. BMC Plant Biology.

[ref-51] Yue FN, Han TY, Zhang YF (2019). Investigation, protection and utilization of the habitat conditions of for the germplasm resources of *Rhododendron henanense* subsp. *lingbaoense* (*Rhl*). Special Economic Animal and Plant.

[ref-52] Zhang M, Chen Y, Nie L, Jin XF, Liao WF, Zhao SY, Fu CH, Yu LJ (2018). Transceiptome-wide identification and screening of WRKY factor involved in the regulation of taxol biosynthesis in *Taxus chinensis*. Scientific Reports.

[ref-53] Zhang Y, Wang L (2005). The WRKY transcription factor superfamily: its origin in eukaryotes and expansion in plants. BMC Evolutionary Biology.

[ref-54] Zhang C, Wang D, Yang C, Kong N, Shi Z, Zhao P, Nan YY, Nie TK, Wang RQ, Ma HL, Chen Q (2017). Genome-wide identification of the potato WRKY transcription factor family. PLOS ONE.

[ref-55] Zhang HY, Zhang LP, Wu SG, Chen YL, Yu DQ, Chen LG (2021). *AtWRKY75* positively regulates age-triggered leaf senescence through gibberellin pathway. Plant Diversity.

[ref-56] Zhao J (2011). Screening of Arabidopsis activation tagging mutants.

[ref-57] Zheng HJ, Hang Y, Wang XH, Li X, Hu T, Tian YH, Zhang MS (2022). Research progress of WRKY transcription factor family in medicinal plants. Plant Physiology Journal.

[ref-58] Zhou XJ, Li JT, Wang HL, Han JW, Zhang K, Dong SW, Zhang YZ, Ya HY, Cheng YW, Sun SS (2022). The chromosome-scale genome assembly, annotation and evolution of Rhododendron henanense subsp. lingbaoense. Molecular Ecology Resources.

[ref-59] Zhou XJ, Wang HL, Li FL, Zhang K, Wang YN, Ya HY (2019). Development of polymorphic SSR markers in *Rhododendron henanense* subsp. *lingbaoense* based on RAD-seq. Journal of Agricultural Biotechnology.

[ref-60] Zhu JQ (2021). Preliminary investigation on the roles of peanut XTHs during seed germination.

