# Supplementary figures and images for: Genome-wide identification and expression analysis of the WRKY gene family in Rhododendron henanense subsp. lingbaoense
Source: PeerJ. 2024 May 29;12:e17435. doi: 10.7717/peerj.17435 (PMC11143974; doi:10.7717/peerj.17435)

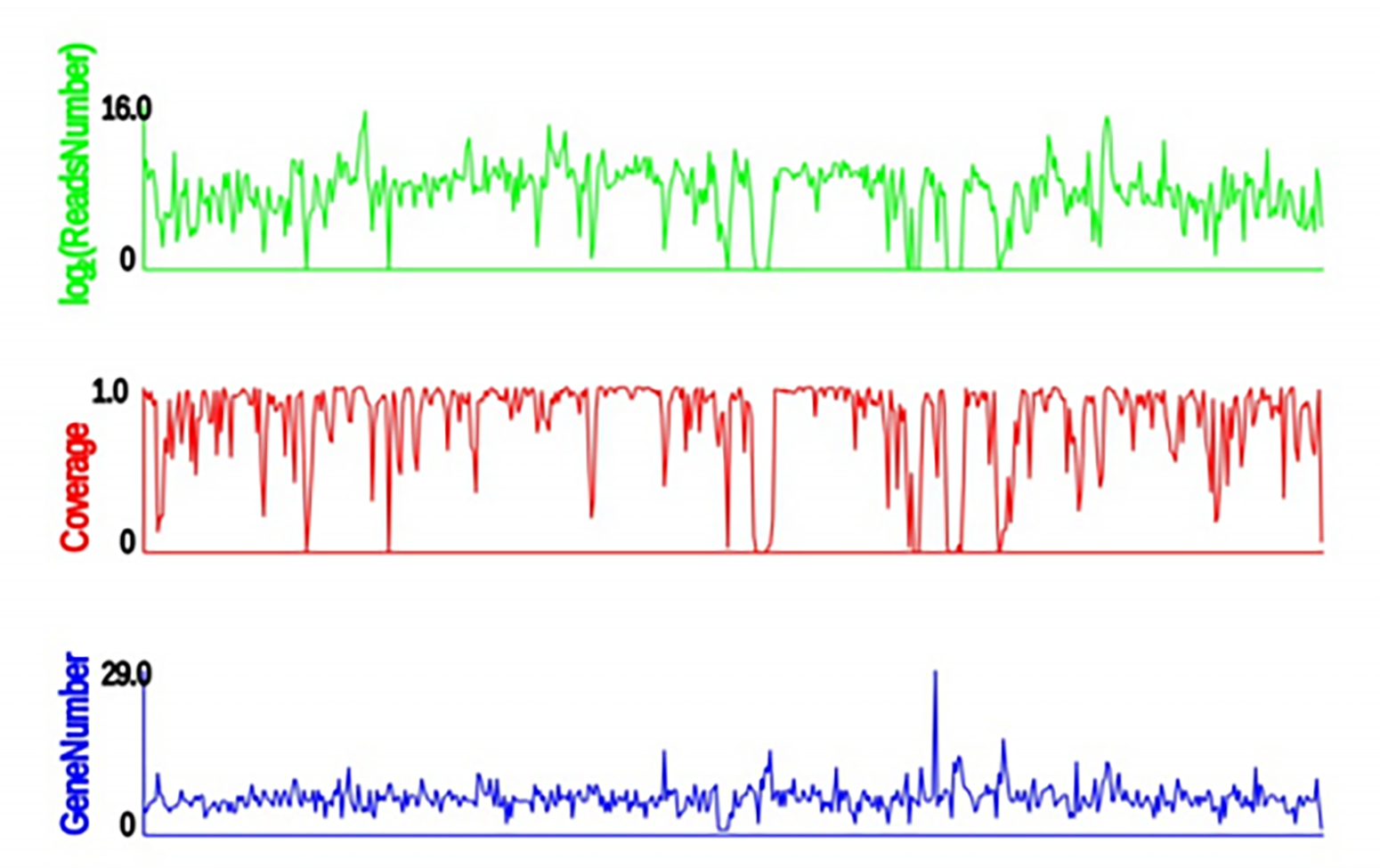

Supplement: Supplemental Information 1 [file peerj-12-17435-s001.png]

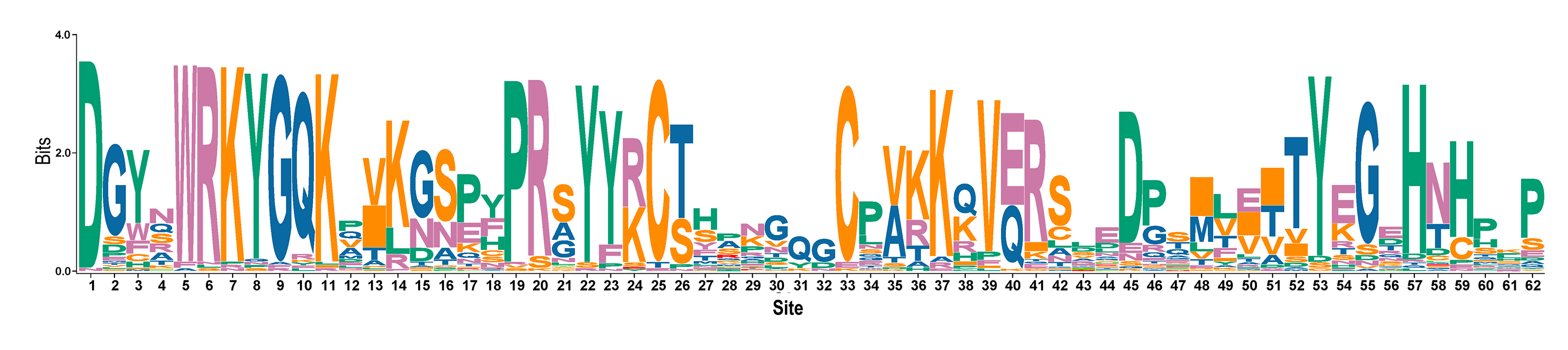

Supplement: Supplemental Information 2 [file peerj-12-17435-s002.png]
