# Supplementary material for: Genome-wide identification and expression analysis of the WRKY gene family in Rhododendron henanense subsp. lingbaoense
Source: PeerJ. 2024 May 29;12:e17435. doi: 10.7717/peerj.17435 (PMC11143974; doi:10.7717/peerj.17435)
Supplement: Supplemental Information 3 — Values of Ks, Ka, and Ka/Ks for Duplicate Gene Pairs [file peerj-12-17435-s003.docx]

**Table S1 RNA Seqpower**

| group | cv | depth | power |
| --- | --- | --- | --- |
| F-vs-H | 0.46170431 | 36 | 0.4088 |
| L-vs-F | 0.501424286 | 36 | 0.3619 |
| L-vs-H | 0.137203872 | 36 | 0.9757 |
| L-vs-R | 0.145851149 | 36 | 0.9695 |
| L-vs-S | 0.1580921 | 36 | 0.9587 |
| R-vs-F | 0.460137017 | 36 | 0.4109 |
| R-vs-H | 0.109918765 | 36 | 0.9891 |
| R-vs-S | 0.131286231 | 36 | 0.9794 |
| S-vs-F | 0.487279847 | 36 | 0.3777 |
| S-vs-H | 0.120393908 | 36 | 0.9850 |

**Table S2. Primer sequences for qPCR**

| **Primer name** | **Primer sequence (5'-3')** |
| --- | --- |
| *RhlWRKY17-F* | AAGACGTGGACCACTCCAAT |
| *RhlWRKY17-R* | AAGGGTCTAAAGTCGCCACA |
| *RhlWRKY19-F* | ATGTCGGTCTCTCCCTCTCT |
| *RhlWRKY19-R* | TTTCCAACCGTTGCTTCGTT |
| *RhlWRKY37-F* | CCAATTCGCATCGCACTTTG |
| *RhlWRKY37-R* | CTTGCACGAGAGGTTCCAAG |
| *RhlWRKY42-F* | TTAGGCCCAAGTGCAACAAC |
| *RhlWRKY42-R* | GCCTTATTCGGAACCCAACC |
| *RhlWRKY45-F* | CAAAGAGCCAAACCGACACA |
| *RhlWRKY45-R* | CTACTGGGCCGGATACCAAA |
| EF1α*-F* | TGTCATCGATGCTCCTGGAC |
| EF1α*-R* | TCTCGGGTCTGACCATCCTT |

**Table S3 Values of Ks, Ka, and Ka/Ks for Duplicate Gene Pairs**

| Gene pair | Duplication events | Ka value | Ks value | Ka/Ks value |
| --- | --- | --- | --- | --- |
| *RhlWRKY_4&RhlWRKY_57* | Tandem replication | 0.0092 | 0.0332 | 0.2759 |
| *RhlWRKY_4&RhlWRKY_58* | Tandem replication | 0.0046 | 0.0221 | 0.2071 |
| *RhlWRKY_57&RhlWRKY_58* | Tandem replication | 0.0046 | 0.0219 | 0.2087 |
| *RhlWRKY_55&RhlWRKY_57* | Tandem replication | 0.2000 | 0.6144 | 0.3256 |
| *RhlWRKY_10&RhlWRKY_30* | Tandem replication | 1.5319 | 1.9786 | 0.7742 |
| *RhlWRKY_9&RhlWRKY_13* | Tandem replication | 1.0092 | 2.4793 | 0.4070 |
| *RhlWRKY_3&RhlWRKY_63* | Tandem replication | 1.4098 | 3.6097 | 0.3906 |
| *RhlWRKY_23&RhlWRKY_25* | Tandem replication | 1.3374 | 3.9851 | 0.3356 |
| *RhlWRKY_14&RhlWRKY_61* | Segmental replication | 0.8392 | 2.9923 | 0.2804 |
| *RhlWRKY_2&RhlWRKY_61* | Segmental replication | 1.2124 | 2.3251 | 0.5214 |
| *RhlWRKY_13&RhlWRKY_21* | Segmental replication | 0.5123 | 2.3488 | 0.2181 |
| *RhlWRKY_14&RhlWRKY_33* | Segmental replication | 0.7518 | 2.4531 | 0.3065 |
| *RhlWRKY_24&RhlWRKY_28* | Segmental replication | 1.5153 | 4.1572 | 0.3645 |
| *RhlWRKY_35&RhlWRKY_37* | Segmental replication | 1.1890 | 3.8546 | 0.3085 |
| *RhlWRKY_39&RhlWRKY_52* | Segmental replication | 0.5165 | 1.9673 | 0.2625 |
| *RhlWRKY_16&RhlWRKY_47* | Segmental replication | 0.8477 | 3.5414 | 0.2394 |
| *RhlWRKY_54&RhlWRKY_59* | Segmental replication | 0.7792 | 2.2334 | 0.3489 |
| *RhlWRKY_12&RhlWRKY_29* | Segmental replication | 1.6695 | 2.0935 | 0.7975 |
| *RhlWRKY_14&RhlWRKY_15* | Segmental replication | 1.2104 | 2.1044 | 0.5752 |
| *RhlWRKY_14&RhlWRKY_17* | Segmental replication | 0.9363 | 3.3053 | 0.2833 |
| *RhlWRKY_14&RhlWRKY_26* | Segmental replication | 0.2530 | 1.6550 | 0.1529 |
| *RhlWRKY_14&RhlWRKY_32* | Segmental replication | 1.4207 | 2.2875 | 0.6211 |
| *RhlWRKY_14&RhlWRKY_59* | Segmental replication | 1.3139 | 1.9550 | 0.6721 |
| *RhlWRKY_14&RhlWRKY_9* | Segmental replication | 0.8122 | 2.3337 | 0.3480 |
| *RhlWRKY_15&RhlWRKY_18* | Segmental replication | 1.8672 | 3.7203 | 0.5019 |
| *RhlWRKY_17&RhlWRKY_18* | Segmental replication | 0.9359 | 1.2251 | 0.7639 |
| *RhlWRKY_18&RhlWRKY_29* | Segmental replication | 1.5287 | 4.2852 | 0.3567 |
| *RhlWRKY_18&RhlWRKY_50* | Segmental replication | 2.0184 | 3.9323 | 0.5133 |
| *RhlWRKY_7&RhlWRKY_18* | Segmental replication | 0.9314 | 2.4683 | 0.3774 |
| *RhlWRKY_2&RhlWRKY_44* | Segmental replication | 0.8208 | 2.5437 | 0.3227 |
| *RhlWRKY_21&RhlWRKY_46* | Segmental replication | 0.5755 | 2.2716 | 0.2533 |
| *RhlWRKY_14&RhlWRKY_24* | Segmental replication | 0.9183 | 3.0472 | 0.3014 |
| *RhlWRKY_21&RhlWRKY_24* | Segmental replication | 1.4680 | 3.8775 | 0.3786 |
| *RhlWRKY_24&RhlWRKY_44* | Segmental replication | 1.3500 | 3.9472 | 0.3420 |
| *RhlWRKY_6&RhlWRKY_24* | Segmental replication | 1.4352 | 4.0866 | 0.3512 |
| *RhlWRKY_7&RhlWRKY_24* | Segmental replication | 0.9214 | 4.2716 | 0.2157 |
| *RhlWRKY_25&RhlWRKY_32* | Segmental replication | 1.3416 | 4.0630 | 0.3302 |
| *RhlWRKY_28&RhlWRKY_30* | Segmental replication | 1.3401 | 3.7874 | 0.3538 |
| *RhlWRKY_6&RhlWRKY_28* | Segmental replication | 0.2636 | 1.0325 | 0.2553 |
| *RhlWRKY_7&RhlWRKY_28* | Segmental replication | 1.3789 | 3.1375 | 0.4395 |
| *RhlWRKY_29&RhlWRKY_51* | Segmental replication | 0.0047 | 0.0114 | 0.4079 |
| *RhlWRKY_29&RhlWRKY_54* | Segmental replication | 0.3533 | 1.9241 | 0.1836 |
| *RhlWRKY_29&RhlWRKY_59* | Segmental replication | 0.4463 | 2.1569 | 0.2069 |
| *RhlWRKY_7&RhlWRKY_29* | Segmental replication | 1.3789 | 3.1375 | 0.4395 |
| *RhlWRKY_22&RhlWRKY_31* | Segmental replication | 0.1787 | 0.6686 | 0.2672 |
| *RhlWRKY_29&RhlWRKY_33* | Segmental replication | 1.3325 | 3.9115 | 0.3407 |
| *RhlWRKY_33&RhlWRKY_50* | Segmental replication | 0.2249 | 0.7591 | 0.2962 |
| *RhlWRKY_33&RhlWRKY_56* | Segmental replication | 0.6039 | 1.7937 | 0.3367 |
| *RhlWRKY_6&RhlWRKY_33* | Segmental replication | 0.6865 | 3.3093 | 0.2074 |
| *RhlWRKY_21&RhlWRKY_34* | Segmental replication | 0.5728 | 2.1421 | 0.2674 |
| *RhlWRKY_34&RhlWRKY_46* | Segmental replication | 0.0027 | 0.0086 | 0.3176 |
| *RhlWRKY_35&RhlWRKY_38* | Segmental replication | 1.1978 | 4.0454 | 0.2961 |
| *RhlWRKY_35&RhlWRKY_43* | Segmental replication | 1.2475 | 4.0471 | 0.3083 |
| *RhlWRKY_35&RhlWRKY_44* | Segmental replication | 1.3836 | 3.9641 | 0.3490 |
| *RhlWRKY_7&RhlWRKY_35* | Segmental replication | 1.3279 | 4.0096 | 0.3312 |
| *RhlWRKY_12&RhlWRKY_36* | Segmental replication | 1.3426 | 4.0581 | 0.3308 |
| *RhlWRKY_13&RhlWRKY_36* | Segmental replication | 0.8561 | 3.4476 | 0.2483 |
| *RhlWRKY_14&RhlWRKY_36* | Segmental replication | 0.1761 | 0.6751 | 0.2609 |
| *RhlWRKY_18&RhlWRKY_36* | Segmental replication | 0.7081 | 2.4238 | 0.2922 |
| *RhlWRKY_21&RhlWRKY_36* | Segmental replication | 1.3670 | 2.6640 | 0.5131 |
| *RhlWRKY_26&RhlWRKY_36* | Segmental replication | 0.3351 | 1.8252 | 0.1836 |
| *RhlWRKY_36&RhlWRKY_47* | Segmental replication | 0.8784 | 2.9743 | 0.2953 |
| *RhlWRKY_36&RhlWRKY_59* | Segmental replication | 0.7984 | 2.8814 | 0.2771 |
| *RhlWRKY_6&RhlWRKY_36* | Segmental replication | 1.2096 | 4.0278 | 0.3003 |
| *RhlWRKY_26&RhlWRKY_37* | Segmental replication | 1.2743 | 3.4243 | 0.3721 |
| *RhlWRKY_37&RhlWRKY_59* | Segmental replication | 1.6380 | 4.3091 | 0.3801 |
| *RhlWRKY_6&RhlWRKY_37* | Segmental replication | 1.2336 | 3.7917 | 0.3253 |
| *RhlWRKY_17&RhlWRKY_38* | Segmental replication | 1.6623 | 2.6401 | 0.6296 |
| *RhlWRKY_4&RhlWRKY_29* | Segmental replication | 1.5398 | 3.5501 | 0.4337 |
| *RhlWRKY_27&RhlWRKY_40* | Segmental replication | 0.1597 | 0.7555 | 0.1597 |
| *RhlWRKY_1&RhlWRKY_42* | Segmental replication | 0.3933 | 1.9204 | 0.3933 |
| *RhlWRKY_38&RhlWRKY_42* | Segmental replication | 0.2738 | 2.4976 | 0.2738 |
| *RhlWRKY_1&RhlWRKY_43* | Segmental replication | 0.4518 | 1.6066 | 0.4518 |
| *RhlWRKY_38&RhlWRKY_43* | Segmental replication | 0.2229 | 1.2751 | 0.2229 |
| *RhlWRKY_29&RhlWRKY_44* | Segmental replication | 1.2315 | 2.5695 | 0.4793 |
| *RhlWRKY_7&RhlWRKY_44* | Segmental replication | 1.8483 | 2.1325 | 0.8667 |
| *RhlWRKY_15&RhlWRKY_47* | Segmental replication | 0.7827 | 3.5428 | 0.2209 |
| *RhlWRKY_2&RhlWRKY_47* | Segmental replication | 0.7625 | 3.4995 | 0.2179 |
| *RhlWRKY_22&RhlWRKY_47* | Segmental replication | 0.5814 | 2.8097 | 0.5814 |
| *RhlWRKY_35&RhlWRKY_47* | Segmental replication | 0.7146 | 3.4661 | 0.2062 |
| *RhlWRKY_44&RhlWRKY_47* | Segmental replication | 1.0307 | 3.5514 | 0.2902 |
| *RhlWRKY_45&RhlWRKY_47* | Segmental replication | 0.2415 | 1.6684 | 0.1448 |
| *RhlWRKY_47&RhlWRKY_49* | Segmental replication | 0.8178 | 3.4838 | 0.2347 |
| *RhlWRKY_47&RhlWRKY_60* | Segmental replication | 0.6258 | 1.9228 | 0.3255 |
| *RhlWRKY_47&RhlWRKY_61* | Segmental replication | 1.0376 | 3.1112 | 0.3335 |
| *RhlWRKY_19&RhlWRKY_52* | Segmental replication | 0.6670 | 1.9980 | 0.3338 |
| *RhlWRKY_29&RhlWRKY_52* | Segmental replication | 1.0932 | 3.6883 | 0.2964 |
| *RhlWRKY_7&RhlWRKY_52* | Segmental replication | 0.9815 | 4.2350 | 0.2318 |
| *RhlWRKY_39&RhlWRKY_53* | Segmental replication | 0.2173 | 0.5393 | 0.4029 |
| *RhlWRKY_52&RhlWRKY_53* | Segmental replication | 0.1986 | 0.5193 | 0.3824 |
| *RhlWRKY_7&RhlWRKY_53* | Segmental replication | 0.5164 | 1.5037 | 0.3434 |
| *RhlWRKY_51&RhlWRKY_54* | Segmental replication | 0.4782 | 2.5335 | 0.1887 |
| *RhlWRKY_51&RhlWRKY_59* | Segmental replication | 0.3708 | 2.1530 | 0.1722 |
| *RhlWRKY_6&RhlWRKY_10* | Segmental replication | 0.2875 | 0.8459 | 0.3399 |
| *RhlWRKY_6&RhlWRKY_32* | Segmental replication | 0.9053 | 3.0476 | 0.2971 |
| *RhlWRKY_18&RhlWRKY_60* | Segmental replication | 2.4881 | 2.2008 | 1.1306 |
| *RhlWRKY_21&RhlWRKY_60* | Segmental replication | 1.6103 | 2.8022 | 0.5747 |
| *RhlWRKY_33&RhlWRKY_60* | Segmental replication | 1.4620 | 3.7546 | 0.3894 |
| *RhlWRKY_37&RhlWRKY_60* | Segmental replication | 0.1691 | 0.6346 | 0.2665 |
| *RhlWRKY_59&RhlWRKY_60* | Segmental replication | 0.6735 | 3.4137 | 0.1973 |
| *RhlWRKY_7&RhlWRKY_60* | Segmental replication | 0.8946 | 2.3500 | 0.3807 |
| *RhlWRKY_13&RhlWRKY_61* | Segmental replication | 1.5142 | 3.8039 | 0.3981 |
| *RhlWRKY_40&RhlWRKY_63* | Segmental replication | 0.3919 | 1.8989 | 0.2064 |
| *RhlWRKY_6&RhlWRKY_63* | Segmental replication | 1.4134 | 3.6898 | 0.3831 |
| *RhlWRKY_7&RhlWRKY_64* | Segmental replication | 1.6017 | 3.5579 | 0.4502 |
