# Supplementary material for: Genome-wide identification and expression analysis of the WRKY gene family in Rhododendron henanense subsp. lingbaoense
Source: PeerJ. 2024 May 29;12:e17435. doi: 10.7717/peerj.17435 (PMC11143974; doi:10.7717/peerj.17435)
Supplement: Supplemental Information 5 [file peerj-12-17435-s005.docx]

**MIQE checklist**

**Table S4. MIQE checklist**

| **ITEM TO CHECK** | **IMPORTANCE** | **CHECKLIST** |
| --- | --- | --- |
| **EXPERIMENTAL DESIGN** |  |  |
| Definition of experimental and control groups | **E** | on page 6, line 107-109 in the manuscript |
| Number within each group | **E** | on page 6, line110 |
| Assay carried out by core lab or investigator's lab? | **D** | on page 7, line 122-129 |
| Acknowledgement of authors' contributions | **D** | on page 20, line 343-348 |
| **SAMPLE** |  |  |
| Description | **E** | on page 6, line 106-108 |
| Volume/mass of sample processed | **D** | on page 6, line 109 SM |
| Microdissection or macrodissection | **E** | on page 6, line 109 |
| Processing procedure | **E** | on page 6, line 107-111 |
| If frozen - how and how quickly? | **E** | SM |
| If fixed - with what, how quickly? | **E** | NA |
| Sample storage conditions and duration (especially for FFPE samples) | **E** | on page 6, line 110 |
| **NUCLEIC ACID EXTRACTION** |  |  |
| Procedure and/or instrumentation | **E** | on page 7, line 122-124. |
| Name of kit and details of any modifications | **E** | on page 7, line 122 |
| Source of additional reagents used | **D** | φ |
| Details of DNase or RNAse treatment | **E** | SM |
| Contamination assessment (DNA or RNA) | **E** | on page 7, line 122-123 |
| Nucleic acid quantification | **E** | on page 7, line 123-124 |
| Instrument and method | **E** | on page 7, line 122-124 |
| Purity (A260/A280) | **D** | SM |
| Yield | **D** | SM |
| RNA integrity method/instrument | **E** | SM |
| RIN/RQI or Cq of 3' and 5' transcripts | **E** | SM |
| Electrophoresis traces | **D** | SM |
| Inhibition testing (Cq dilutions, spike or other) | **E** | **φ** |
| **REVERSE TRANSCRIPTION** |  |  |
| Complete reaction conditions | **E** | **SM** |
| Amount of RNA and reaction volume | **E** | **SM** |
| Priming oligonucleotide (if using GSP) and concentration | **E** | **SM** |
| Reverse transcriptase and concentration | **E** | **SM** |
| Temperature and time | **E** | **SM** |
| Manufacturer of reagents and catalogue numbers | **D** | **SM** |
| Cqs with and without RT | **D*** | **Not determined** |
| Storage conditions of cDNA | **D** | **SM** |
| **qPCR TARGET INFORMATION** |  |  |
| efficiency and LOD of each assay. | **E** | **NA** |
| Sequence accession number | **E** | on page 7, line 134 |
| Location of amplicon | **D** | φ |
| Amplicon length | **E** | SM |
| *In silico* specificity screen (BLAST, etc) | **E** | on page 5, line86-88 |
| Pseudogenes, retropseudogenes or other homologs? | **D** | on page 13, line207-208 |
| Sequence alignment | **D** | φ |
| Secondary structure analysis of amplicon | **D** | φ |
| Location of each primer by exon or intron (if applicable) | **E** | NA |
| What splice variants are targeted? | **E** | NA |
| **qPCR OLIGONUCLEOTIDES** |  |  |
| Primer sequences | **E** | on page 7, line125 in the manuscript (Table S2) |
| RTPrimerDB Identification Number | **D** | NA |
| Probe sequences | **D**** | NA |
| Location and identity of any modifications | **E** | NA |
| Manufacturer of oligonucleotides | **D** | on page 7, line126 |
| Purification method | **D** | φ |
| **qPCR PROTOCOL** |  |  |
| Complete reaction conditions | **E** | SM |
| Reaction volume and amount of cDNA/DNA | **E** | SM |
| Primer, (probe), Mg^2+^ and dNTP concentrations | **E** | φ |
| Polymerase identity and concentration | **E** | φ |
| Buffer/kit identity and manufacturer | **E** | φ |
| Exact chemical constitution of the buffer | **D** | φ |
| Additives (SYBR Green I, DMSO, etc.) | **E** | SM |
| Manufacturer of plates/tubes and catalog number | **D** | SM |
| Complete thermocycling parameters | **E** | SM |
| Reaction setup (manual/robotic) | **D** | SM |
| Manufacturer of qPCR instrument | **E** | SM |
| **qPCR VALIDATION** |  |  |
| Evidence of optimisation (from gradients) | **D** | OK |
| Specificity (gel, sequence, melt, or digest) | **E** | SM |
| For SYBR Green I, Cq of the NTC | **E** | NA |
| Standard curves with slope and y-intercept | **E** | SM |
| PCR efficiency calculated from slope | **E** | SM |
| Confidence interval for PCR efficiency or standard error | **D** | SM |
| r2 of standard curve | **E** | SM |
| Linear dynamic range | **E** | SM |
| Cq variation at lower limit | **E** | SM |
| Confidence intervals throughout range | **D** | SM |
| Evidence for limit of detection | **E** | SM |
| If multiplex, efficiency and LOD of each assay. | **E** | NA |
| **DATA ANALYSIS** |  |  |
| qPCR analysis program (source, version) | **E** | SM |
| Cq method determination | **E** | SM |
| Outlier identification and disposition | **E** | SM |
| Results of NTCs | **E** | on page 7, line130 |
| Justification of number and choice of reference genes | **E** | on page 7, line126-127 in the manuscript (Table S2). |
| Description of normalisation method | **E** | SM |
| Number and concordance of biological replicates | **D** | on page 7, line129 |
| Number and stage (RT or qPCR) of technical replicates | **E** | on page 7, line129 |
| Repeatability (intra-assay variation) | **E** | φ |
| Reproducibility (inter-assay variation, %CV) | **D** | φ |
| Power analysis | **D** | φ |
| Statistical methods for result significance | **E** | on page 29, line523 |
| Software (source, version) | **E** | on page 7, line130-131 |
| Cq or raw data submission using RDML | **D** | see SupplementaryFile3raw data.rar |

Note: SM Supplementary material; Φ Information is not available; NA Not applicable; OK Test was performed but evidence is missing. supplementary_File3_rawdata.rar

**The details are as follows:**

**EXPERIMENTAL DESIGN**

1.Definition of experimental and control groups. on page 6, line 107-109 in the manuscript.

Control group: 2-month-old seedlings without any treatment, i.e. 0 control.

Experimental groups: Two-month-old seedlings were subjected to treatments with low-temperature (4 ℃), 20% PEG, NaCl (200 mM), and MeJA (200 µM) solutions for 1, 3, 6, and 12 hours.

2. Number within each group. on page 6, line 110 in the manuscript.

There were 5 samples in the low-temperature (4 ℃), 20% PEG, NaCl (200 mM), and MeJA (200 µM) solutions group respectively.

3. Assay carried out by core lab or investigator's lab? on page 7, line 122-129 in the manuscript.

Assay carried out by investigator's lab (the Molecular Biology Laboratory of Luoyang Normal University).

**SAMPLE**

1.Description. on page 6, line 106-108 in the manuscript.

Seeds of Rhl were collected from Xiaoqinling, Lingbao City, Henan Province. The seeds were decontaminated, sterilized and cultured on MS medium at 25 ℃ (16 hours light/8 hours dark).

Volume/mass of sample processed. SM.

100mg of sample processed.

Microdissection or macrodissection. on page 6, line 109 in the manuscript.

Macrodissection.

2. Processing procedure. on page 6, line 107-111 in the manuscript.

If frozen - how and how quickly? SM.

Liquid nitrogen is quickly frozen for about 20 seconds and then stored at -80 ℃.

Sample storage conditions and duration (especially for FFPE samples). on page 6, line 109 in the manuscript.

Sample stored at -80 ℃ for at least three months.

**NUCLEIC ACID EXTRACTION**

1.Procedure and/or instrumentation. on page 7, line 122-124.

Name of kit and details of any modifications. on page 7, line 122 in the manuscript.

RNA was extracted using a magnetic bead total RNA kit from Shanghai Lingjun Biotechnology Co., Ltd.

Modification: Due to the high content of polysaccharides and polyphenols in *Rhododendron henanense* subsp. *lingbaoense*, 5% DTT and 3% PK were added to the lysis solution before extraction.

2. Details of DNase or RNAse treatment. SM.

The sample magnetic beads cleaned by WB1 were added with an appropriate amount of DNase working liquid, placed at room temperature for 20-30 minutes, the supernatant was discarded, and then continued to be cleaned with WB2.

3. Contamination assessment (DNA or RNA). on page 7, line 122-123 in the manuscript.

RNA contamination was assessed as follows:

OD230: absorbance of impurities (phenols, glycogen and other carbohydrates);

OD260: absorbance of nucleic acid;

OD280: absorbance of protein;

OD260/280: The ratio of pure RNA is between 1.5 and 2.1, if the ratio is low, it indicates that it is contaminated by protein;

OD260/230: The ratio of pure RNA is between 2.0 and 2.5, if the ratio is low, it indicates that it is polluted by organic matter such as sugar, peptide, phenol, etc.

Electrophoresis was used to identify the integrity of RNA.

4.Nucleic acid quantification. on page 7, line 123-124 in the manuscript.

Instrument and method. on page 7, line 122-124 in the manuscript.

The total RNA samples were extracted using the plant magnetic bead method kit (Shanghai Lingjun Biotechnology Co., Ltd). The RNA concentration and OD value (A_260_/A_280_) were measured using an ultra micro spectrophotometer (NanoDrop One, Thermo Scientific).

Purity (A260/A280) and Yield. SM**.**

| Processing mode | Processing time/h | Nucleic acid detection | ng/μl | A260/A280 | A260/A230 |
| --- | --- | --- | --- | --- | --- |
| Contrast | 0 | RNA | 117.8 | 1.72 | 1.59 |
| MEJ | 1 | RNA | 89.6 | 1.76 | 1.69 |
| MEJ | 3 | RNA | 85.5 | 1.62 | 1.68 |
| MEJ | 6 | RNA | 90.8 | 1.64 | 1.64 |
| MEJ | 12 | RNA | 80.6 | 1.70 | 1.68 |
| 4℃ | 1 | RNA | 87.3 | 1.73 | 1.72 |
| 4℃ | 3 | RNA | 91.5 | 1.68 | 1.65 |
| 4℃ | 6 | RNA | 90.0 | 1.70 | 1.68 |
| 4℃ | 12 | RNA | 89.6 | 1.59 | 1.61 |
| PEG | 1 | RNA | 95.4 | 1.68 | 1.67 |
| PEG | 3 | RNA | 100.5 | 1.75 | 1.64 |
| PEG | 6 | RNA | 118.2 | 1.81 | 1.70 |
| PEG | 12 | RNA | 90.5 | 1.76 | 1.75 |
| NaCl | 1 | RNA | 95.2 | 1.78 | 1.72 |
| NaCl | 3 | RNA | 86.8 | 1.68 | 1.65 |
| NaCl | 6 | RNA | 92.3 | 1.70 | 1.68 |
| NaCl | 12 | RNA | 90.6 | 1.73 | 1.70 |

Note: The results in the table above are the average of the three times.

5.RNA integrity method/instrument. SM.

Intact RNA usually has three bands, the brightest being the 28S band, followed by the 18S band, and the lightest being the 5S band (some kits filter out the 5S band during extraction). The ratio of 28S to 18S can be measured by 1% agarose gel electrophoresis. Electrophoresis apparatus and electrophoresis tank are used by China Beijing 61 Company. This method is mainly used to detect the purity and integrity of RNA.

RIN/RQI or Cq of 3' and 5' transcripts.

| Processing mode/time | 0 | 1 | 3 | 6 | 12 |
| --- | --- | --- | --- | --- | --- |
| MEJ | 9.0 | 9.1 | 8.7 | 8.3 | 7.6 |
| 4℃ |  | 7.5 | 8.0 | 7.8 | 9.0 |
| PEG |  | 6.5 | 7.6 | 6.8 | 8.7 |
| NaCl |  | 8.6 | 6.9 | 7.8 | 9.0 |

Note: The results in the table above are the average of the three times.

Electrophoresis traces. SM.


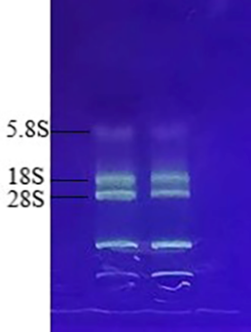


Note: Partial representative results (left 0 control, right PEG treatment 1h)

6. Inhibition testing (Cq dilutions, spike or other). Φ.

**REVERSE TRANSCRIPTION.**

1.Complete reaction conditions.SM.

This RNA was used to synthesize cDNA by reverse transcription, with using the TOYOBO ReverTra Ace qPCR RT Master Mix(Code No. FSQ-201). (1) Denaturation of RNA: 65 ℃ denaturation 5 min; (2) Reaction system(20 µL): Nuclease-free Water 11 µL, 5×RT Buffer 4 µL, RT Enzyme Mix 1 µL, Primer Mix 1 µL, RNA 3 µL. (3) Reverse transcription: 37 ℃ 15min, 98℃ 5min.

**SM** **Supplementary material.**

| Processing mode | Processing time/h | Nucleic acid detection | ng/μl | A260/A280 | A260/A230 |
| --- | --- | --- | --- | --- | --- |
| Contrast | 0 | cDNA | 1888 | 1.93 | 2.11 |
| MEJ | 1 | cDNA | 1760 | 1.94 | 2.15 |
| MEJ | 3 | cDNA | 1758 | 1.93 | 2.14 |
| MEJ | 6 | cDNA | 1848 | 1.94 | 2.11 |
| MEJ | 12 | cDNA | 1688 | 1.94 | 2.17 |
| 4℃ | 1 | cDNA | 1750 | 1.90 | 2.11 |
| 4℃ | 3 | cDNA | 1860 | 1.97 | 2.16 |
| 4℃ | 6 | cDNA | 1800 | 1.96 | 2.13 |
| 4℃ | 12 | cDNA | 1788 | 1.90 | 2.16 |
| PEG | 1 | cDNA | 1780 | 1.98 | 2.10 |
| PEG | 3 | cDNA | 1863 | 1.94 | 2.06 |
| PEG | 6 | cDNA | 1880 | 1.94 | 2.12 |
| PEG | 12 | cDNA | 1746 | 1.86 | 2.04 |
| NaCl | 1 | cDNA | 1830 | 1.83 | 2.01 |
| NaCl | 3 | cDNA | 1720 | 1.84 | 2.05 |
| NaCl | 6 | cDNA | 1800 | 1.90 | 2.04 |
| NaCl | 12 | cDNA | 1748 | 1.92 | 2.01 |

Note: The results in the table above are the average of the three times.

Amount of RNA and reaction volume.SM.

Reaction system (20 µL), RNA 3 µL.

Priming oligonucleotide (if using GSP) and concentration

Primer Mix 1 µL

Reverse transcriptase and concentration. SM.

RT Enzyme Mix 1 µL

Temperature and time. SM.

Reverse transcription: 37 ℃ 15min, 98℃ 5min.

Manufacturer of reagents and catalogue numbers.SM.

The TOYOBO ReverTra Ace qPCR RT Master Mix(Code No. FSQ-201) in Japan.

2. Storage conditions of cDNA.SM.

cDNA stored at -80 ℃

**qPCR TARGET INFORMATION**

1.efficiency and LOD of each assay.NA.

2.Sequence accession number. on page 7, line 134 in the manuscript.

3.Location of amplicon. Φ.

Amplicon length. SM.

| Gene ID | *RhlWRKY17* | *RhlWRKY19* | *RhlWRKY37* | *RhlWRKY42* | *RhlWRKY45* |
| --- | --- | --- | --- | --- | --- |
| Amplicon length | 129bp | 153bp | 129bp | 194bp | 142bp |

In silico specificity screen (BLAST, etc). on page 5, line86-88 in the manuscript.

The protein sequences of 90 WRKY genes from Arabidopsis were obtained from the PlantTFDB to generate the criteria to search the *Rhl* genome database using BlastP. A total of 65 putative WRKY genes were identified.

Pseudogenes, retropseudogenes or other homologs? on page 13, line207-208 in the manuscript.

*RhlWRKY_56* and *_65* were not detected in any of the five tissues, suggesting these might be pseudogenes.

Sequence alignment. Φ.

Secondary structure analysis of amplicon. Φ.

4.Location of each primer by exon or intron (if applicable). NA.

What splice variants are targeted? NA .

**qPCR OLIGONUCLEOTIDES**

1. Primer sequences. on page 7, line125 in the manuscript (Table S1).

| *RhlWRKY17-F* | AAGACGTGGACCACTCCAAT |
| --- | --- |
| *RhlWRKY17-R* | AAGGGTCTAAAGTCGCCACA |
| *RhlWRKY19-F* | ATGTCGGTCTCTCCCTCTCT |
| *RhlWRKY19-R* | TTTCCAACCGTTGCTTCGTT |
| *RhlWRKY37-F* | CCAATTCGCATCGCACTTTG |
| *RhlWRKY37-R* | CTTGCACGAGAGGTTCCAAG |
| *RhlWRKY42-F* | TTAGGCCCAAGTGCAACAAC |
| *RhlWRKY42-R* | GCCTTATTCGGAACCCAACC |
| *RhlWRKY45-F* | CAAAGAGCCAAACCGACACA |
| *RhlWRKY45-R* | CTACTGGGCCGGATACCAAA |
| EF1α*-F* | TGTCATCGATGCTCCTGGAC |
| EF1α*-R* | TCTCGGGTCTGACCATCCTT |

2.RTPrimerDB Identification Number. NA.

3.Probe sequences. NA.

4.Location and identity of any modifications. NA.

5.Manufacturer of oligonucleotides. on page 7, line126 in the manuscript.

Sangon Biotech (Shanghai) Co., Ltd.

6.Purification method. Φ Information is not available.

**qPCR PROTOCOL. SM.**

1.Complete reaction conditions: Reactions were prepared at room temperature.

Reaction volume and amount of cDNA/DNA: 10 µL 2×SuperReal PreMix Plus, 2µL primer, 2 µL 5 × diluted cDNA, 6µL Nuclease-free ddH_2_O.

Primer, (probe), Mg^2+^ and dNTP concentrations: Primers at 4 µM, Φ.

Polymerase identity and concentration: Platinum *Taq* DNA Polymerase, Φ.

Buffer/kit identity and manufacturer: SM. SYBR GreenER^TM^ Two-Step qRT-PCR Kit Universa.

Exact chemical constitution of the buffer: Φ.

Additives (SYBR Green I, DMSO, etc.): SM. SYBR GreenER^TM^.

2.Manufacturer of plates/tubes and catalog number: SM. Kirgen Research 0.2 mL PCR Tubes, Cat, no.221040H.

3.Complete thermocycling parameters: SM. The total volume of the reaction system was 20 μL, and the cycle conditions included 95℃ for 2 min, 95℃ for 10 s, 60℃ for 30 s, 72℃ for 30 s for 41 cycles, 95℃ for 1 min, 55℃ for 1 min, and then the melt curve analysis was performed at 55℃ for 10 s and 98℃ for 5 s.

Reaction setup (manual/robotic): SM. CFX96Real-Time PCR software Robot.

Manufacturer of qPCR instrument: SM. Bio-Rad CFX96Real-Time PCR Detection System.

**qPCR VALIDATION**

1.Evidence of optimisation (from gradients) : OK.

2.Specificity (gel, sequence, melt, or digest). SM.

Melt curve analysis was performed at 55℃ for 10 s and 98℃ for 5 s.

3.For SYBR Green I, Cq of the NTC: SM Cq of the NTC is 0.

4.Standard curves with slope and y-intercept. SM.

PCR efficiency calculated from slope. SM

| Genes | *RhlWRKY17* | *RhlWRKY19* | *RhlWRKY37* | *RhlWRKY42* | *RhlWRKY45* | *EF1α* |
| --- | --- | --- | --- | --- | --- | --- |
| Efficiency | 1.98 | 1.91 | 1.95 | 1.94 | 1.93 | 1.88 |

Confidence interval for PCR efficiency or standard error.SM.

Confidence interval for PCR efficiency is 102%.

r^2^ of standard curve: SM r^2^ is 0.9986.

5.Linear dynamic range. Φ

Cq variation at lower limit. NA.

Evidence for limit of detection. NA.

6.If multiplex, efficiency and LOD of each assay. NA.

**DATA ANALYSIS**

1.qPCR analysis program (source, version). SM.

The qPCR analysis program is Bio Red CFX96Real-Time PCR software from Bole Company.

Cq method determination. SM

Manual positioning above background signal.

Outlier identification and disposition.SM.

The range of Cq values was 15-35, and the Cq value was less than 15, indicating that the amplification was within the baseline period and did not reach the fluorescence threshold. Ideally, there is a linear relationship between the Cq value and the logarithm of the starting copy number of the template, which is the standard curve. According to the standard curve, when the amplification efficiency is 100%, the computed Cq value of single copy number of gene is about 35. If it is greater than 35, the theoretical template starting copy number is less than 1, which can be considered meaningless. The difference between three parallel Cq values should not be greater than 1. If it is greater than 1, it will be done again. If there is a significant difference between one value and the other two, it can be deleted.

2.Results of NTCs. on page 7, line130.

Results of NTC was not detected. The diagram below the represents gene of the Melt peak and Melt Curve. The following figure represents the genes of the melting peak and melting curve.


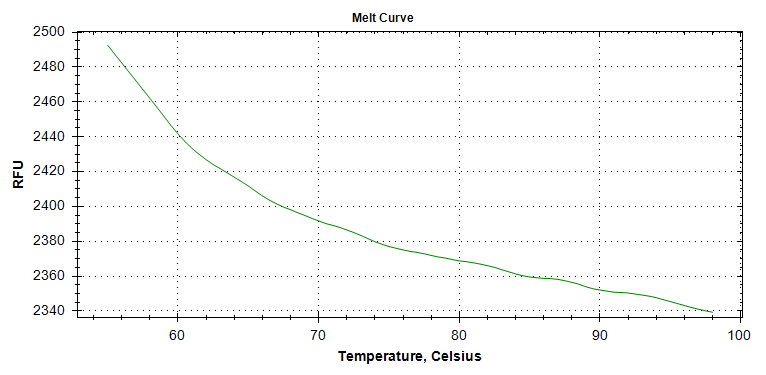

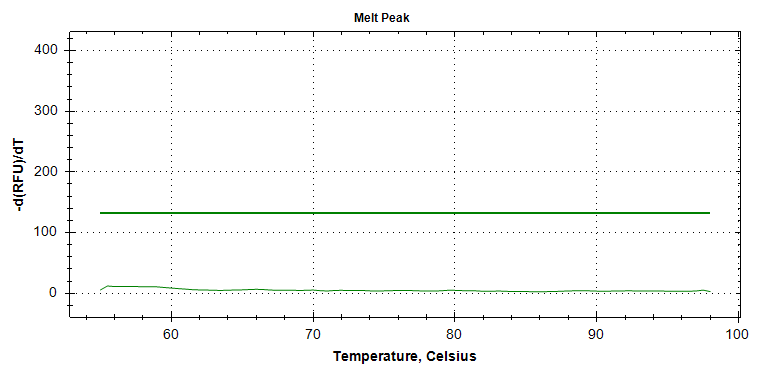

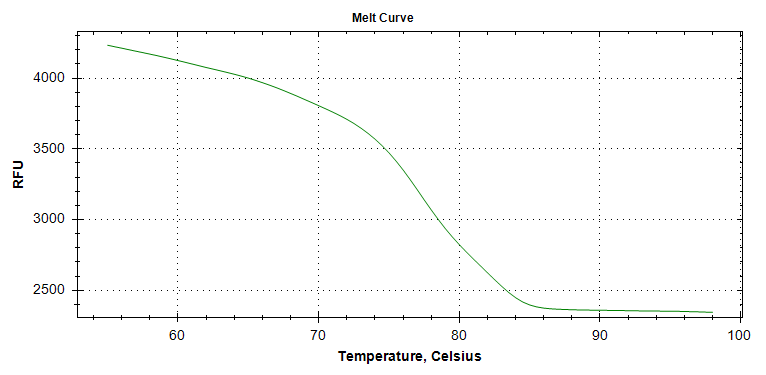

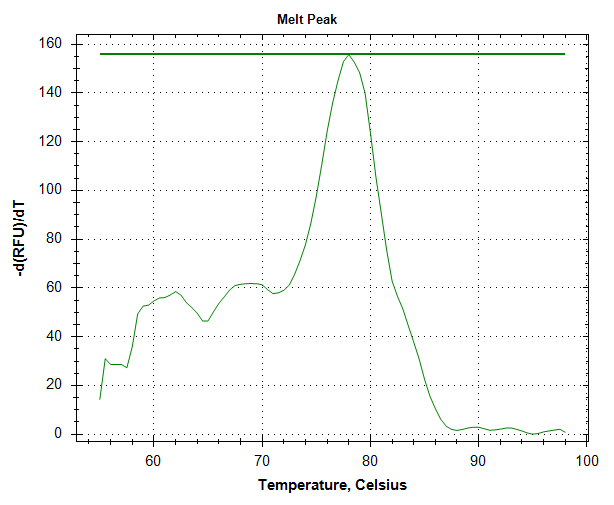


Wrky42

Wrky42

EF1α

EF1α

3.Justification of number and choice of reference genes. on page 7, line125 in the manuscript (Table S1).

Reference genes is *EF1α*(one).

4.Description of normalisation method. SM.

(1) The standard product was diluted into different concentrations and used as a template for PCR reaction.

(2) The standard curve is drawn with the logarithm of the standard copy number as the horizontal coordinate and the measured CT value as the vertical coordinate.

(3) The copy number of the sample can be obtained in the standard curve according to the Cq value of the unknown sample.

5.Number and concordance of biological replicates. on page 7, line129 in the manuscript.

Three biological replicates.

6.Number and stage (RT or qPCR) of technical replicates. on page 7, line129 in the manuscript.

Three technical replicates.

7.Repeatability (intra-assay variation). Φ.

8.Reproducibility (inter-assay variation, %CV). Φ.

9.Power analysis. Φ.

10. Statistical methods for result significance. on page 28, line523 in the manuscript.

Statistical analysis is performed using an one-way analysis of variance.

11. Software (source, version). on page 7, line130-131 in the manuscript.

Single-factor analysis of variance and graphing were performed using Graphpad Prism 8.3 software.

12.Cq or raw data submission using RDML.

For the submission of Cq or raw data, see see Supplementary_File3raw data.rar.
